# Chemiresistor Devices for Chemical Warfare Agent Detection Based on Polymer Wrapped Single-Walled Carbon Nanotubes

**DOI:** 10.3390/s17050982

**Published:** 2017-04-28

**Authors:** John F. Fennell, Hitoshi Hamaguchi, Bora Yoon, Timothy M. Swager

**Affiliations:** 1 Department of Chemistry, Massachusetts Institute of Technology, Cambridge, MA 02139, USA; jfennell@mit.edu (J.F.F.); borayoon@mit.edu (B.Y.); 2 Japanese Synthetic Rubber Company, Tokyo 105-8640, Japan; Hitoshi_Hamaguchi@jsr.co.jp

**Keywords:** conjugated polymers, chemical warfare agents, carbon nanotubes, chemiresistors, sensors

## Abstract

Chemical warfare agents (CWA) continue to present a threat to civilian populations and military personnel in operational areas all over the world. Reliable measurements of CWAs are critical to contamination detection, avoidance, and remediation. The current deployed systems in United States and foreign militaries, as well as those in the private sector offer accurate detection of CWAs, but are still limited by size, portability and fabrication cost. Herein, we report a chemiresistive CWA sensor using single-walled carbon nanotubes (SWCNTs) wrapped with poly(3,4-ethylenedioxythiophene) (PEDOT) derivatives. We demonstrate that a pendant hexafluoroisopropanol group on the polymer that enhances sensitivity to a nerve agent mimic, dimethyl methylphosphonate, in both nitrogen and air environments to concentrations as low as 5 ppm and 11 ppm, respectively. Additionally, these PEDOT/SWCNT derivative sensor systems experience negligible device performance over the course of two weeks under ambient conditions.

## 1. Introduction

Intelligence surveillance and reconnaissance efforts in the military arena benefit from increasing the number of sensors on the battlefield. Placing networked chemical sensors on individual soldiers without increasing an already onerous soldier-load would exponentially surge the information density to reduce uncertainty and the “fog of war” [1]. Responsive, selective, low-energy and low-cost are essential characteristics of such systems. Current military chemical warfare agent (CWA) sensors are sensitive to CWAs to the low ppb level, selective and networkable; but they are cumbersome, expensive, energy and training intensive [2]. These unfavorable characteristics may be mitigated in the future by using employing chemiresistive devices that can be deployed in large numbers, inexpensively and networked together [3]. Chemiresistive devices utilizing single-walled carbon nanotubes (SWCNTs), with their unique mechanical and electrical properties, offer a promising platform to meet these requirements and are excellent substrates on which to build CWA sensors for use on the modern battlefield [4,5,6,7].

There are many established ways to functionalize SWCNTs to impart selectivity for different analytes. Previous reports have used covalent-sidewall functionalization [8,9,10,11,12,13], defect group functionalization [14], non-covalent exohedral functionalization [15,16,17,18,19] (with polymer, surfactants, or composite mixtures) and endohedral functionalization [20,21]. Non-covalent exohedral functionalization with conjugated polymers was chosen for this study because the native conductivity of SWCNTs is preserved without disruption of the π-electronic states in the nanotube sidewalls. We chose to develop polymer-wrapped composites for this sensing application in lieu of a simple, physical mixture because physical mixtures containing SWCNTs phase segregate as a result of strong interactions between the nanotubes that allow them to aggregate. Favorable π-π stacking interactions between the SWCNTs and polymers disrupt this aggregation and allow for processing and the inclusion of polymer originated molecular recognition groups [22,23].

**Figure 1:**
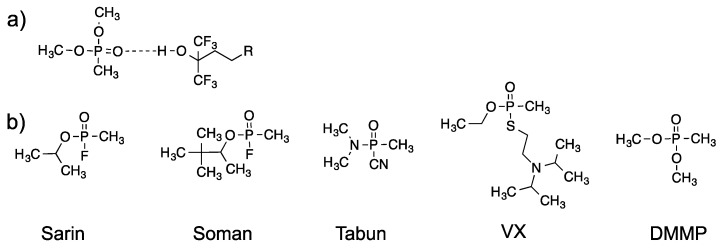
(**a**) Hydrogen bonding interaction between HFIP and DMMP; (**b**) Structures of CWAs and a CWA simulant, DMMP.

Our group has previously demonstrated that composites of SWCNTs and polythiophenes (PTs) modified with a hexafluoroisopropyl group (HFIP) make effective chemiresistive material for a CWA simulant, dimethyl methylphosphonate (DMMP) sensing [24]. Shown in Figure 1a, the HFIP group exhibits a strong hydrogen bonding interaction with phosphate esters [25], which are a common structural component of nerve agents, including sarin, soman, tabun and VX (Figure 1b) [26]. 

**Figure 2:**
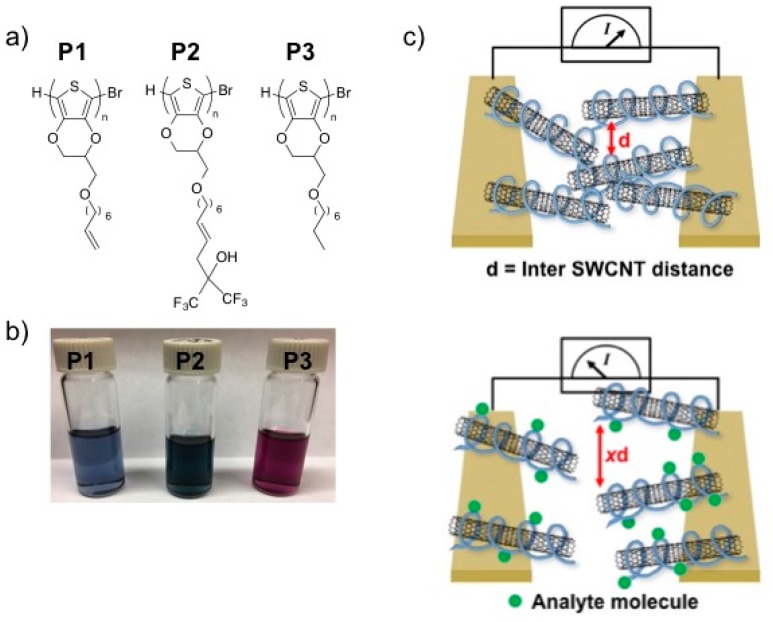
(**a**) Structures of derivatized PEDOTs; (**b**) Photograph of dispersions of **P1**–**P3**/SWCNT dispersions in THF (**P1**, **P3**) and DMF (**P2**); (**c**) Schematic of the swelling transduction mechanism.

Building upon our experience, we report here a sequel to our previous method by using a derivatized poly(3,4-ethylenedioxythiophene) (PEDOT) polymer to produce a more robust CWA sensor. Though the fabricated PT/SWCNT devices proved to be selective and sensitive (sub ppm detection limit), they experienced significant performance degradation within days of fabrication, likely due to SWCNT aggregation. PEDOT, in comparison to PT, is much more polarizable and an electron-rich material, which generates stronger interactions to form a polymer-SWCNT composite with improved stability [27,28]. HFIP groups are introduced to impart selectivity for binding of DMMP and are introduced through the functionalization of terminal alkene-containing sidechain attached to the PEDOT backbone. Alkene cross metathesis reactions [29] facilitate the attachment of molecular recognition elements to the side chains to create derivatized PEDOT polymers with desired selectivity. In this report, we detail three PEDOT analogs, **P1**, **P2** and **P3**, to wrap SWCNTs to create CWA sensitive chemiresistive sensors (Figure 2a). The designed PEDOT derivatives also impart the necessary solubility to effectively de-bundle and disperse SWCNTs as shown in Figure 2b. In contrast to our previous work, where thin films were spin-casted upon a glass substrate, these chemiresistor devices are fabricated by drop casting of a polymer/SWCNT dispersion between two metal electrodes on a glass substrate to form random networks of polymer-wrapped SWCNTs (Figure 2c, top). Using this drop-casting methodology, a very small amount of polymer/SWCNT is used to make a large number of devices. 

**P2** is functionalized with a pendant HFIP group on the side chain that is known to form strong hydrogen bonds with phosphonates. This interaction concentrates the selected analytes proximate to the SWCNT. The conductance is then modulated through a combination of mechanisms including directly affecting transport along a given SWCNT by charge transfer to change the doping level [30,31] or electrostatic interactions with the positive charge characters through dipolar induced pinning or scattering [32]. Inter-SWCNT transport can also be modulated by the binding of analytes to the HFIP group on the polymer sidechains and an associated swelling increases the inter-SWCNT distance to create wider tunnel junctions between SWCNTs that lower the conductivity [4]. A cartoon demonstrating the latter mechanism is drawn in Figure 2c. In this report we demonstrate CWA selective chemiresistors based upon a **P2**/SWCNT composition and reveal strong responses upon exposure to low concentrations (5 ppm) of the nerve agent simulant DMMP. We also demonstrate a fairly reliable performance of the **P2**/SWCNT sensor in the presence of air containing 24% relative humidity (RH). Furthermore, we explore the effects of device aging under ambient conditions. In addition, our experiments confirm that EDOT derivatives create a versatile platform polymer for the development of polymer wrapped SWCNT chemiresistors. 

## 2. Materials and Methods

### 2.1. Materials

All chemicals and reagents were purchased from Sigma-Aldrich and used without additional purification, except that tetrahydrofuran was distilled from sodium metal and benzophenone. SWCNTs (6,5 chirality, carbon (95%), with 93% as SWCNTs) were acquired from Sigma Aldrich, Inc. (Saint Louis, MO, USA). 3,4-dimethoxythiophene (95%) and 2-(allyl)hexafluoroisopropanol (99%) were purchased from Matrix Scientific (Columbia, SC, USA). 1,3-bis(diphenylphosphino)propane nickel (II) (99%) was purchased from Strem Chemicals (Newburyport, MA, USA). Deuterated solvents for NMR were obtained from Cambridge Isotope Laboratories (Tewksbury, MA, USA).

### 2.2. Characterization Methods

^1^H, ^13^C and ^19^F NMR spectra were recorded at 400 MHz (100 MHz) or 500 MHz (125 MHz) using either Bruker AVANCE-400 or JEOL JNM-ECZR-500 NMR spectrometers, respectively. Chemical shifts are reported in ppm and referenced to residual NMR solvent peaks (^1^H NMR: δ 3.62 ppm for THF, δ 7.26 ppm for CDCl_3_; ^13^C NMR: δ 77.2 ppm for CDCl_3_). High-resolution mass spectra were determined with a Bruker Daltonics APEXIV 4.7 Tesla FT–ICR–MS using ESI or DART ionization. The MALDI-MS spectra were acquired in linear and reflection modes in the Koch Institute at MIT using a Bruker Microflex MALDI-MS spectrometer. UV–Vis absorption spectra were measured using an Agilent Cary 4000 Series UV–Vis spectrophotometer. Gel permeation chromatography (GPC) measurements were performed in tetrahydrofuran using an Agilent 1260 Infinity system and calibrated with a polystyrene standard. ATR–FTIR spectra were acquired using a Thermo Scientific Nicolet 6700 FT–IR with either a Ge or ZnSE crystal for ATR and subjected to the ‘atmospheric suppression’ correction in OMNIC™ Spectra software. Raman spectra were collected with excitation at 633 nm laser using a Horiba LabRAM HR800 Raman spectrometer. 

### 2.3. Synthesis and Characterization of Monomers and Polymers

**Scheme 1:**
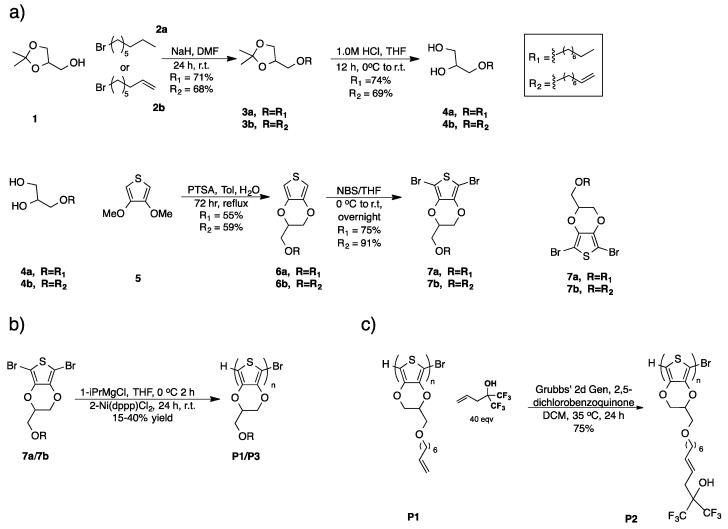
(**a**) Synthesis of derivatized EDOT monomers for (**b**) Kumada catalyst transfer polycondensation (**c**) Post-polymerization modification via alkene cross-metathesis of **P1** and allyl-HFIP to form **P2**.

Detailed procedures for the synthesis of all compounds can be found in the supplementary information. Briefly, brominated EDOT monomers can be polymerized using Kumada catalyst transfer polycondensation (KCTP) [33,34,35,36,37,38]. The monomer synthesis begins by O-alkylation of the inexpensive reagent solketal (**1**) to yield compounds **3a** and **3b** in moderate yields (Scheme 1). Intermediates **4a** and **4b** were next obtained by acid-catalyzed deprotection in a mixture of 1.0 M HCl (aq) and THF. Monomer precursors **6a** and **6b** were formed by an acid-catalyzed substitution of the methoxy groups of compound **5** by diols **4a** and **4b**. Products **6a** and **6b** were then brominated with *N*-bromosuccinnamide (NBS) in THF to provide KCTP monomers **7a** and **7b**.

KCTP was chosen as the polymerization method to generate our derivatized PEDOT derivatives over electrochemical polymerization [39,40] and FeCl_3_-catalyzed oxidative polymerization [41]. Though oxidative polymerizations do not often yield cross-linked polymer products in the preparation of polythiophenes, in this case, both of these methods yielded insoluble products that appeared to be highly cross-linked. We hypothesize that the terminal alkene on the side chain of **P1** reacts with the radical cation intermediates generated under oxidizing conditions to give cross-linked products.

**P1** is, in principle, a platform polymer upon which we can incorporate a number of desired recognition elements. **P2** contains a HFIP moiety that was attached via a cross metathesis reaction [13,29,42,43]. As a result of its unique hydrogen bonding characteristics, HFIP is a known selector for DMMP (Figure 1). **P3** was studied as a control polymer. **P1** and **P3** were synthesized using KCTP, which were then solvent fractionated to isolate the highly soluble material. Such soluble fractions were produced in acceptable yields (15–40%) (Scheme 1b), with M_n_ values of 2700 to 2900 g/mol and polydispersities of 1.2–1.3 (Figure S7). Although higher molecular weight fractions with masses up to 6100 g/mol were identified using MALDI-MS for **P1** (Figure S8), higher molecular weight samples were found to be only slightly soluble in organic solvents and unable to form composites with SWCNTs. For **P1**–**P3**, we observed that molecular weights determined by MALDI MS were smaller than that of GPC [44]. Additionally, molecular weights determined by end group analysis with ^1^H NMR are higher than GPC or MALDI MS results (Table S1 and Figures S8–S11). The lower molecular weights obtained by MALDI MS analysis as compared to GPC is consistent with previous work that demonstrated GPC tends to overestimate molecular weights of rod-like conjugated systems [44]. It is possible that MALDI-MS analysis provides smaller molecular weights than ^1^H NMR end group analysis because the higher laser power required to volatize higher molecular weight fractions leads to chain fragmentation [45]. 

**P2** was synthesized via a post-polymerization modification of **P1** using an alkene cross metathesis reaction (Scheme 1c). This transformation was successful, as evidenced by an increase in molecular weight from 2900 to 3900 g/mol. The conversion of a terminal alkene in **P1** to an internal alkene in **P2** was confirmed by analysis of the ^1^H NMR spectrum. The disappearance of a doublet at δ 5.78 ppm and multiplet centered at δ 4.96 ppm in **P1** (Figure S12) to yield a doublet at δ 5.40 ppm in **P2** indicates full conversion of the terminal alkene to an internal olefin (Figure S13). The intensity of both peaks in this doublet suggests an approximately equal proportion of both *cis* and *trans* stereoisomers are present in **P2**. In Figure S14, ^19^F NMR confirms the presence of a HFIP group in **P2** with a resonance at δ 76.7 ppm. 

### 2.4. Preparation of Polymer/SWCNT Dispersions

To a solution of **P1**(**P3**) (M_n_ = 2900 g/mol, 10 mg) in dry tetrahydrofuran (THF, 5 mL) or **P2** in dry dimethyl formamide (DMF, 5 mL), 5 mg of SWCNT was added and followed by 10 µL hydrazine (a reducing/de-doping agent). Then the resulting mixture was sonicated for 1 h in an ultrasonic bath (Branson, 3510) chilled with ice and then allowed to reach room temperature. Subsequently, the suspension was centrifuged for 30 min at 9500 g. The supernatant was decanted and centrifuged again for an additional 30 min at 9500 g and allowed to stand overnight undisturbed. The isolated supernatant was directly used for the device fabrication via dropcasting unless otherwise indicated. For UV–vis–NIR absorption spectroscopy, the isolated supernatant was diluted to 1:2 in THF or DMF, further sonicated for 5 min, and recorded in a 1 cm optical path quartz cuvette.

### 2.5. Preparation of Gold Electrodes on Glass Microscope Slides

Glass substrates deposited with chromium adhesion layers (10 nm) and gold electrodes (100 nm) were prepared according to a literature procedure [17]. Briefly, glass slides (VWR Microscope Slides) were cleaned by sonication in acetone for 5 min followed by UV-ozone treatment using a UVO cleaner (Jelight Company Inc., Model 42, Irvine, CA, USA), for 20 min. A 10 nm layer of chromium (99.99%, R.D. Mathis) and a subsequent 100 nm layer of gold (99.99%, R.D. Mathis) were deposited through a custom stainless steel mask using a thermal evaporator (Angstrom Engineering, Kitchener, ON, Canada), which resulted in three sets of four channel electrode patterns on a single glass slide, which was cut into three individual devices. Each device contains a gold pattern of four isolated working electrodes with one shared reference-counter electrode on the glass substrate. The gap between one pair of gold electrodes is 1 mm.

### 2.6. Fabrication of a Polymer-SWCNT Chemiresistor Platform

The desired amount of polymer-SWCNT dispersion in THF or DMF was dropcasted with a 20 μL syringe between four gold electrode pairs on the glass substrate. Typically, between 4–20 μL of the dispersion was required to reach the target electrode resistance of 10–50 kΩ. 

### 2.7. Volatile Organic Compound (VOC) Gas Detection Measurement

For VOC gas detection measurement, the fabricated array device was placed into a custom-built PTFE enclosure with a small gas inlet and outlet, the gold electrodes of the device were connected to a PalmSens EmStat potentiostat with a MUX16 multiplexer. A KIN-TEK gas generator system calibrated for each VOC was used to deliver to the device’s enclosure a known concentration of a given VOC analyte diluted in N_2_ gas at a fixed gas flow rate (200 mL/min) to minimize drift in the baseline resistance. For calibration, emission rate of each VOC by monitoring the decrease in mass of each liquid for an hour at a 200 mL/min flow rate and a designated temperature (40 °C–70 °C). Three trials for each VOC were performed. The potentiostat applied a constant potential of 0.1 V across the electrodes, and the current for each channel of the device was recorded using PS Trace software (v. 4.7) during 60 s of VOC vapor exposures. After a linear baseline correction, the change in current resulting from exposure to the analyte was converted to the negative change in conductance (−Δ*G*/*G*_0_ (*%*) = (*I*_0_ − *I*)/*I*_0_) × 100), where *I*_0_ is initial current), which was taken as the device’s response. Schematics and photographs of the experimental setup can be found in the Supporting Information, Figure S39 (Schematic/cartoon for sensing experimental set up), Figure S40 (Photograph of sensing experimental set up) and Figure S41 (Schematic/cartoon for sensing experimental set up in humid air).

## 3. Results and Discussion

### 3.1. Dispersion of SWCNTs with Functionalized PEDOTs

Our goal was to create polymer/SWCNT dispersions that are resistant to re-bundling to be able to reproducibly create durable devices. Dispersed and de-bundled nanotubes have increased surface area for interaction with analytes and can create conductive pathways that can be more readily disrupted by analyte binding as is preferred for sensing applications [16]. We generated our derivatized polymer/SWCNT dispersions after experimentally determining the ratios of polymer to SWCNTs in the dispersions that produced the highest chemiresistive responses to DMMP. We identified the optimal ratio by weight of polymer/SWCNT/solvent was 2/1/1 (see Figure S16). 

**Figure 3:**
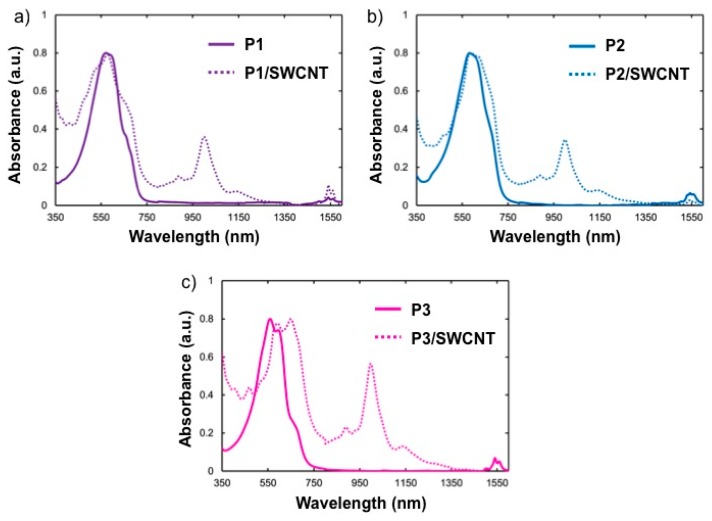
UV–vis/NIR spectra of polymer/SWCNT dispersions. (**a**) **P1**/SWCNT; (**b**) **P2**/SWCNT; (**c**) **P3**/SWCNT.

Figure 3a–c presents UV–vis–NIR absorbance spectra of **P1**–**P3** overlayed with the spectra of their respective diluted SWCNT dispersions. The absorption spectrum suggests the presence of both semiconducting and metallic nanotubes. Absorption bands in the 800 to 1600 nm as well as the 550 to 900 nm region are indicative of the E_11_ and E_22_ van Hove singularity transitions of semiconducting carbon nanotube, while the transitions of metallic nanotubes can be found in the region of 400–600 nm [46,47]. The clarity and resolution in these bands in Figure 3 indicate the presence of de-bundled SWCNTs in the polymer/SWCNT dispersions. All compositions have some broad absorptions in the 400–600 nm region, indicating the presence of metallic SWCNTs. The resolved absorption bands located at 880, 990, and 1120 nm are evidence of the presence of (6,5) SWCNTs [48]. It may be inferred that **P3** (Figure 3c) is more effective at dispersing SWCNTs than **P1** and **P2** from inspection of the intensity ratios of the SWCNT-based absorbances at 990 nm relative to that associated with **P3** (λ_max_ = 553 nm). The features at 1550 nm are attributed to a vibrational overtone from hydrazine [49], which is used to prevent oxidation of the dispersions and stabilize de-bundled state. 

**Figure 4:**
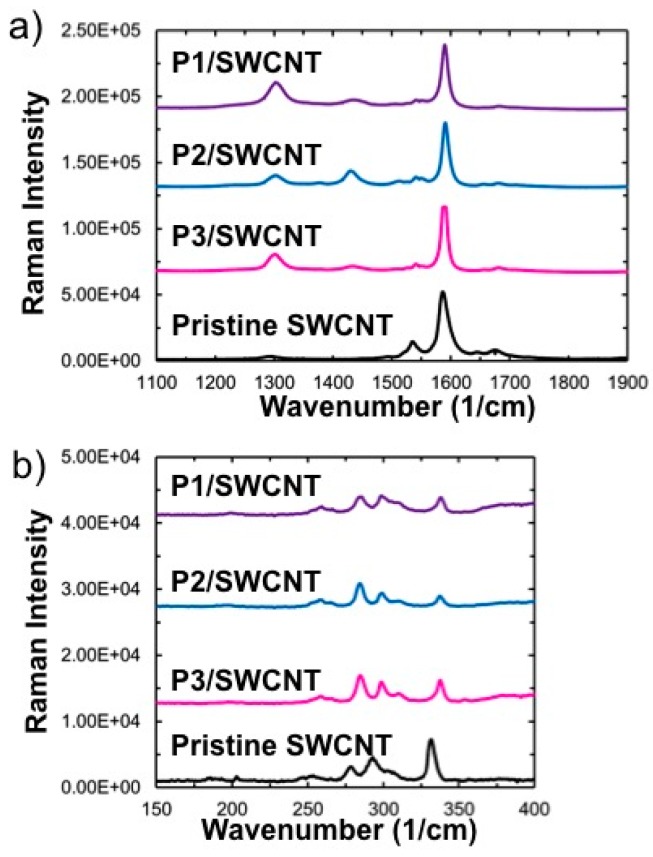
Resonance Raman spectra (truncated) of polymer/SWCNT dispersions with focus on the (**a**) D & G band region (1200–1700 cm^−1^) and the (**b**) RBM region (100–300 cm^−1^).

Resonance Raman spectroscopy is a useful tool in confirming the presence of SWCNTs in polymer dispersions. The full resonance Raman spectra of the composites and pure polymer are given in Figures S17 and S18. Thin film samples were prepared for analysis by drop casting dispersions from THF (**P1** and **P3**) or DMF (**P2**) onto a silicon wafer. A pristine SWCNT thin film was prepared by drop casting (6,5) enriched SWCNTs from a fresh dispersion in *ortho*-dichlorbenzene on to a silicon wafer. The spectra were taken using a 633 nm excitation wavelength and are normalized to the intensity of the G-band, at 1590 cm^−1^ and offset for clarity. In Figure 4a, Raman spectrum for the D-G band region is displayed. The D-band, located at 1290 cm^−1^, is indicative of the disruption of the sp^2^ network in conjugated nanocarbon systems [50]. The ratio of intensities of the D band (1290 cm^−1^), to the G band (*I_D_*/*I_G_*) can give relative measure of disruption of the graphene π-system. After dispersion, there is a large increase in *I_D_*/*I_G_* from the pristine SWCNTs to the polymer-dispersed SWCNTs. Specifically, the *I_D_*/*I_G_* is 0.06 in pristine SWCNTs and 0.9, 0.8, and 0.7 for the **P1**/SWCNT, **P2**/SWCNT and **P3**/SWCNT composites, respectively. This indicates an increase in the number of defects in the SWCNT sidewalls within the dispersion [50]. The source of the disruption in this case is hypothesized to be breaking of the conjugation of the SWCNT sp^2^ network during the sonication step in the dispersion preparation or the result of strong associations between the graphene walls and the dispersing polymers or other molecules/molecular fragments [51,52]. The broad Raman peak located at 1422 cm^−1^ in the polymer dispersions is attributed entirely to the polymer and can be seen distinctly in Figure S18. The radial breathing modes (RBM), shown in Figure 4b, are the signature for the presence of carbon nanotubes and are located between 100–300 cm^−1^. The peak frequencies are inversely proportional to tube diameter. The as received pristine SWCNTs gave a peak at 252 cm^−1^ and overlapping peaks at 277 cm^−1^ and 290 cm^−1^, whereas the polymer/SWCNT dispersions are slightly narrowed maxima and positioned at 283 cm^−1^ and 297 cm^−1^. A full Raman spectrum is shown in Figure S17.

### 3.2. Polymer/SWCNT Composites for DMMP Detection in Dry N_2_ and Air (24% RH)

**Figure 5:**
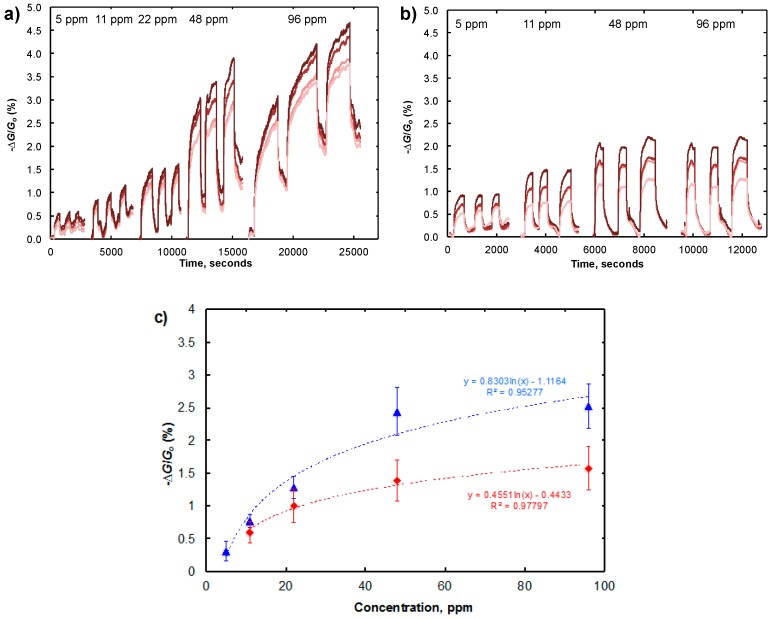
(**a**) Conductance traces of four **P2**/SWCNT-based chemiresistors to five concentrations of DMMP in dry N_2_ at room temperature; (**b**) Conductance traces of four **P2**/SWCNT-based chemiresistors to four concentrations of DMMP in air (24% RH) at room temperature; (**c**) Saturated chemiresistive responses averaged across quadruplicate trials for **P2**/SWCNT devices for varying amounts of DMMP in dry N_2_ (blue triangles) and air (24% RH) (red diamonds) at room temperature.

With stable composites in hand, we sought to investigate the efficacy of our polymer/SWCNT composite system in detecting the nerve agent simulant, DMMP. The sensory response was investigated by measuring the change in current between two electrodes at a constant bias voltage of 0.10 V. The change in current was converted to a negative change in conductance [−∆*G*/*G*_0_ (%) = ((*I*_0_ − *I*)/*I*_0_) × 100%], where *I*_0_ is the initial current. This normalized response allows for small differences in the resistivity that can complicate device to device comparisons. Figure 5a shows baseline-corrected responses of four chemiresistive devices that were tested in parallel incorporating our HFIP-PEDOT/SWCNT system (**P2**/SWCNT) that is designed to selectively detect DMMP. Figure 5a shows the response in dry N_2_, while Figure 5b shows the response in air with 24% RH. Exposure times were varied to reach full saturation of the devices. In both carrier gases, the sensors show a reversible response across this concentration range. We do observe increased baseline drift in the N_2_ conductance trace in contrast to the more stable baseline in the air trace. We suggest that the stability is due to the steady presence of water and oxygen at the device interface that acts to maintain consistent electronic environment in the sensor material. In Figure S42, the device response becomes saturated in a shorter time period at all concentrations in air than in N_2_. In Figure 5c, we observed a linear responses range for DMMP from 5–48 ppm in dry N_2_ and air. The dynamic range extends to 98 ppm, but the response curve suggests saturation behavior of the detector at or near 48 ppm. We noted a 0.30% response to DMMP at a concentration of 5 ppm with a linear response up to 48 ppm for the **P2**/SWCNT response in dry N_2_. We calculated a 2.7 ppm detection limit in dry N_2_ using a signal to noise ratio method [53] for **P2**/SWCNT. While we could not detect DMMP at a concentration of 5 ppm in air with 24% RH, we observed a 0.60% response to DMMP at a concentration of 11 ppm with a linear response up to 48 ppm for the **P2**/SWCNT. The calculated detection limit for **P2**/SWCNT in air is 6.5 ppm. Figure S43 demonstrates the linear response curve for experiments in N_2_ and air. The **P2**/SWCNT composite device covers an operationally relevant range for the detection of nerve agents such as sarin (GB), for which DMMP is a simulant [54]. The chemiresistive detection of DMMP by a decrease in conductance is consistent with the notion of a transduction mechanism that relies upon a swelling of the SWCNT network or direct modulation of the SWCNT conductance by charge transfer of dipolar mechanisms. These effects are all consistent with the DMMP being concentrated in the polymer HFIP-terminated side chain as a result of hydrogen-bonding interactions with the **P2**/SWCNT composite (Figure 1a). Uptake of the analyte DMMP into the SWCNT network increases the resistance (reduces the current) as a result.

**Figure 6:**
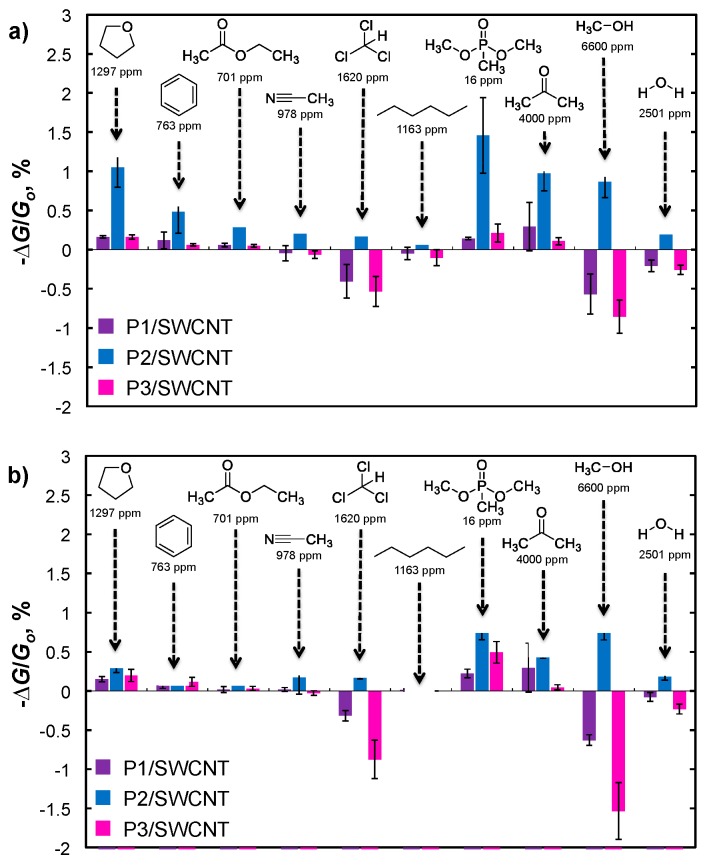
(**a**) Chemiresistive responses averaged across four **P1**–**P3**/SWCNT devices to 60 s exposures of VOCs at a flow rate of 200 mL/min in dry N_2_; (**b**) Chemiresistive responses averaged across four **P1**–**P3**/SWCNT devices to 60 s exposures of VOCs at a flow rate of 200 mL/min in air (24% RH).

Detecting CWAs in a real-world environmental sample demands a strategy for detecting trace quantities in a complex background containing other vapors that may be present in higher concentrations. Figure 6a shows chemiresistive responses for devices **P1**–**P3**/SWCNTs exposed to various volatile organic chemicals (VOCs) for 60 s followed by a 140 s recovery time at a constant flow rate of 200 mL/min at room temperature in dry N_2_. Figure 6b shows results for a similar experiment obtained by using air (24% RH). The concentrations of vapors selected to confound the sensors for this experiment were chosen to be sufficiently high to obtain a measureable response from pristine SWCNTs (Figures S19 and S19a–c). In both dry N_2_ and air, it is clear that the **P2**/SWCNT composite responds to polar and hydrogen bonding analytes to a much greater degree than the HFIP free reference composites (**P1**/SWCNT and **P3**/SWCNT). The enhanced response is therefore attributed to presence of the polar and proton donating HFIP group of **P2**.

The **P2**/SWCNT composite responds to all analytes in N_2_ and air tested but exhibits exceptional selectivity for DMMP above all others in dry N_2_. Vapor challenges of 1297 ppm THF, 4000 ppm acetone and 6600 ppm MeOH all have similar magnitude responses (around 1.0%) to what is observed for 16 ppm DMMP in N_2_. In air, the responses of all **P1**–**P3**/SWCNT are slightly lower than in dry N_2_. We hypothesize that the introduction of water at lower concentrations into the gas mixture offers competition as a hydrogen bond donor to DMMP. Therefore, the presence of water decreases the amount of DMMP that may be bound to the **P2**/SWCNT composite, thus lowering the chemiresistive response.

**Figure 7:**
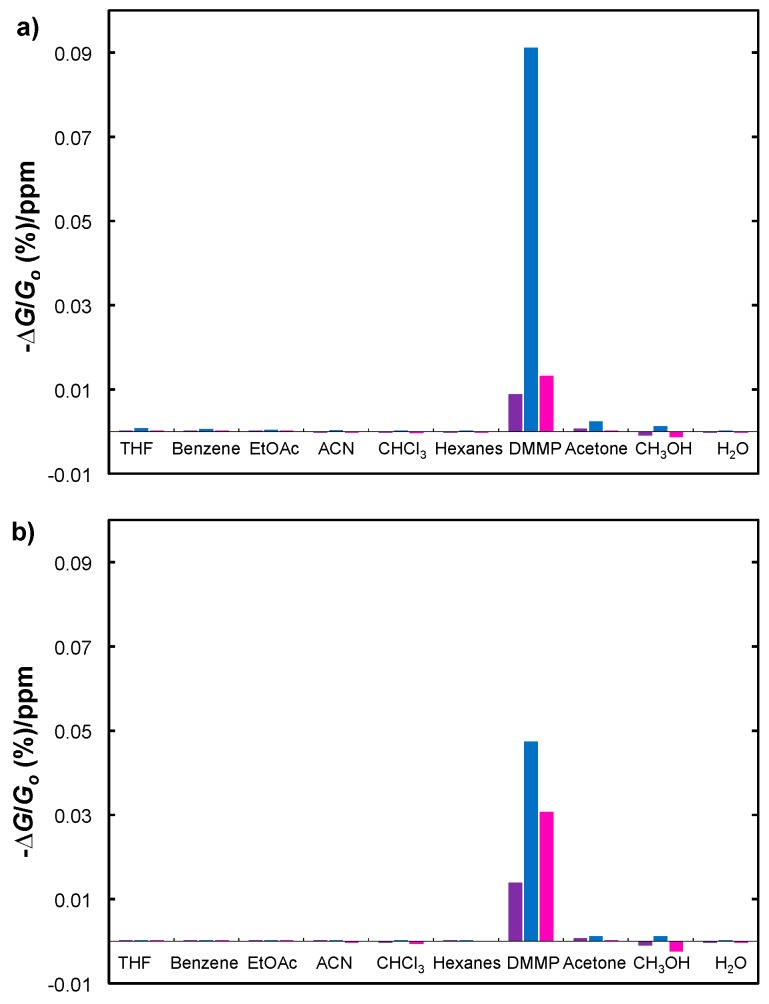
(**a**) Chemiresistive response ratio (−∆*G*/*G*_0_ (%)/ppm) for **P2**/SWCNT to VOCs in N2; (**b**) Chemiresistive response ratio for **P2**/SWCNT to VOCs in air (24% RH).

It is clear that the **P2**/SWCNT is selective as designed in N_2_ and air. To put this selectivity for DMMP in perspective, we have provided a response ratio by dividing the negative change in conductance by the concentration in ppm [(−∆*G*/*G*_0_)/ppm] at the given flow rate (200 mL/min). Figure 7b shows that the **P2**/SWCNT response ratio for DMMP in N_2_ is an order of magnitude larger than that for the **P1**/SWCNT and **P3**/SWCNT composites. A similar, though smaller response ratio is shown for **P2**/SWCNT in air. The **P2**/SWCNT composite garners a nearly a two orders of magnitude larger response ratio to DMMP over the other VOCs. An interesting result in Figure 6a,b show an increase in conductance for the **P1**/SWCNT and **P3**/SWCNT composites upon exposure to acetonitrile, chloroform, hexanes, methanol and water. This may be explained by a secondary doping effect [55]. Considering that the SWCNTs are naturally *p*-doped by molecular oxygen [56] and that the polymers are strong electron donors, the carrier levels in the SWCNTs will be reduced by strong charge transfer interactions or electron donation from the polymers to nanotubes. Some of the solvents may reduce these interactions and thereby affect an increase in carrier density. If the organic vapor molecules do not have strong interactions with the SWCNTs that pin or scatter the carriers, increased carrier levels will give rise to an increased conduction as we observe. 

To establish the stability of the **P2**/SWCNT composites, we conducted a device aging study in which we measured the chemiresistive response to exposure to 11 ppm DMMP in N_2_ over the course of two weeks (Figure S44). These devices were stored under ambient lab conditions on the benchtop. We found no significant change in the response over that time period. However, though not recorded, we did observe that devices stored under ambient conditions over the course of a more than a month experienced significant degradation in performance. We also tested the stability of the devices in humid air (50% RH). The results in Figure S45 show a slightly enhanced and reliable response at 11 ppm to this level of humidity with a response of 0.95%. As mentioned before, the response in air (24% RH) was 0.60%. While the presence of water at lower RH served to compete with the **P2**/SWCNT composite for hydrogen bonding with DMMP, at higher RH, there likely is water available in the gas mixture to compensate hole carriers in the *p*-type SWCNTs, thus reducing conductance. This observation of decreasing conductance of SWCNTs with increasing humidity has been reported previously [57,58] and is linear until high RH (>65% are reached). It will continue be important in future SWCNT-based device design to take into account the effect of humidity.

## 4. Conclusions

In summary, we developed a chemiresistive sensor for the detection of nerve agent simulant DMMP. This sensor was fabricated using a derivatized PEDOT/SWCNT composite and with strong responses to 5 ppm DMMP and a calculated detection limit of 2.7 ppm in N_2_. The same devices in a “real world” environment (air with 24% RH), experienced a strong response at 11 ppm and a detection limit of 6.5 ppm. We explored the effects on the devices of aging in an ambient environment and higher humidity (50% RH). Additionally, we propose that the transduction mechanism responsible for the decrease in conductance in the device upon the introduction of DMMP is a hydrogen bonding interaction between the DMMP and an HFIP moiety incorporated in the derivatized PEDOT sidechain. Finally, we demonstrated that our polymer/SWCNT composites maintain stable, debundled dispersions in solution via resonance Raman and UV–vis–NIR spectroscopy. 

## Supplementary Materials

The following are available online at http://www.mdpi.com/1424-8220/17/5/982/s1, Synthetic procedures for preparation of monomers and polymers, molecular weight analysis (GPC, MALDI-MS), ^1^H, ^19^F and ^13^C NMR spectra, FTIR-ATR spectra, full range resonance Raman spectra, and additional sensing data.
