# Supplementary material for: Chemiresistor Devices for Chemical Warfare Agent Detection Based on Polymer Wrapped Single-Walled Carbon Nanotubes
Source: Sensors (Basel). 2017 Apr 28;17(5):982. doi: 10.3390/s17050982 (PMC5469335; doi:10.3390/s17050982)

Supplementary Information for

Chemiresistor Devices for Chemical Warfare Agent Detection Based on Polymer Wrapped Single-Walled Carbon Nanotubes

John F. Fennell Jr., Hitoshi Hamaguchi, Bora Yoon, Timothy M. Swager

Department of Chemistry, Massachusetts Institute of Technology

77 Massachusetts Avenue, Cambridge, Massachusetts 02139

tswager@mit.edu

Contents

1. General

2. Synthesis of monomers

3. Synthesis of polymers

4. Molecular weight analysis

5. ^{1}H and ^{13}C NMR spectra of monomers and polymers

6. MALDI-TOF of polymer materials

7. Full range resonance Raman spectroscopy spectra of polymer/SWCNT composites and polymers

8. FTIR-IR spectra of polymer and polymer-SWCNT composites

9. Additional sensing data

1. **General**

All air- and water-sensitive syntheses were performed in flame-dried flasks under an inert atmosphere with dry argon using standard Schlenk techniques. Diethyl ether, tetrahydrofuran, dichloromethane, and toluene were taken from an Innovative Technologies solvent purification system containing activated alumina columns and stored under argon over 3Å or 4Å molecular sieves. Reaction progress was monitored by thin layer chromatography (Merck silica gel 60 F254 plates), and column chromatography was performed using silica gel (60Å pore size, 230–500 mesh, Aldrich).

2. **Synthesis of monomers**

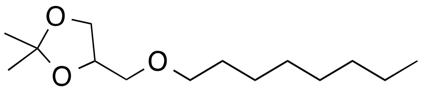

Compound **3a**

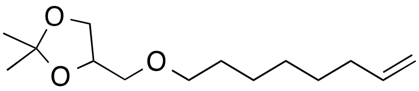

Compound **3b**

Figure S1. Compounds **3a** and **3b**

2,2-dimethyl-4-((octyloxy)methyl)-1,3-dioxolane, (**3a**) and 2,2-dimethyl-4-((oct-7-en-1-yloxy)methyl)-1,3-dioxolane (**3b**) were prepared in the same manner. A typical preparation for **3b** follows. To a flamed-dried 500 mL round bottom flask (RBF) containing 200 mL *N, N*-dimethyl formamide (DMF), 1,2-isopropylideneglycerol (**1**) (23.65 g, 179 mmol) and octenyl bromide (**2b**) (28.5 g, 149 mmol) were delivered. Sodium hydride (5.37 g, 224 mmol) was added over the course of one hour in six equal portions. The reaction was monitored with thin layer chromatography (TLC) using a solution of hexanes:ethyl acetate (90/10%) as the eluent. TLC plates were visualized with a $KMnO_4$ stain. The mixture was stirred at 40 ^{o}C under $Ar_{(g)}$ for 14 hours. The reaction was poured over 1.0 L of a saturated solution of ammonium chloride. The crude mixture was extracted three times with ethyl acetate, washed with brine and 10% solution of LiCl. After drying over $MgSO_4$, the solvent was evaporated under vacuum. The crude product was purified via vacuum distillation to yield 24.7 grams (68.3 % yield) of a clear, viscous liquid (**3b**).
3a characterization: ^{1}H NMR (400 MHz, $CDCl_3$): δ 5.76 (m, 1H), 4.97 (m, 1H), 4.90 (d, 1H), 4.24 (m, 1H) , 4.03 (m, 1H), 3.70 (m, 1H), 3.44 (m, 4H), 2.02 (m, 2H), 1.55 (m, 2H), 1.40 (s, 3H), 1.34 (s, 3H) . ^{13}C NMR (125 MHz, $CDCl_3$) δ 109.5, 74.9, 72.0, 71.9, 67.0, 31.9, 29.7, 29.5, 29.4, 26.9, 26.2, 25.5, 22.8, 14.2 HR-MS (ESI) calc for $C_{14}H_{28}O_3$ $[M+Na]^+$ 267.19, found 267.19. FT-IR (ATR, ν, cm^{-1}) 2991 (w), 2933 (m), 2860 (m), 1682 (w), 1468 (m), 1371 (m), 1216 (m), 1118 (m), 1058 (m), 979 (m), 849 (m), 823 (w), 728 (w)

3b characterization: ^{1}H NMR (500 MHz, $CDCl_3$): δ 7.52−7.42 (m, 6H), 2.03 (t, J = 6.8 Hz, 4H), 1.95 (m, 4H), 1.33−1.26 (m, 8H), 1.00−0.94 (m, 4H). ^{13}C NMR (125 MHz, $CDCl_3$) δ 139.1, 114.3, 109.4, 74.8, 71.9, 67.0, 33.8, 29.6, 29.0, 28.9, 26.8, 26.0, 25.5. HR-MS (ESI) calc for $C_{14}H_{26}O_3$ $[M+H]^+$ 243.1955, found _243.1927. FT-IR (ATR, ν, cm^{-1}) 3096 (w), 2992 (w), 2936 (m), 2864 (m), 1692 (w), 1643 (w), 1464 (w), 1261 (m), 1218 (m), 1056 (s), 912 (m), 849 (m), 823 (w), 735 (w).

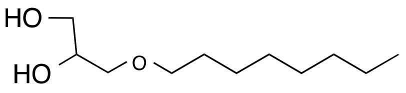

Compound **4a**

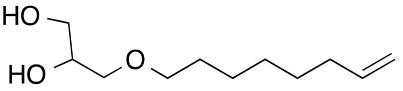

Compound **4b**

Figure S2. Compounds **4a** and **4b**

3-(octyloxy)propane-1,2-diol, **4a**, and 3-(oct-7-en-1-yloxy)propane-1,2-diol, **4b**, were prepared in the same manner. A typical preparation for **4b** follows. To a 1000-mL RBF equipped a magnetic stir bar, **3b** (24.0 g, 99 mmol) and

400 mL of THF were added. Then, 280 mL of 1.0 M $HCl_{(aq)}$ was delivered. The mixture was stirred for 12 hours at room temperature. The reaction was quenched with 280 mL de-ionized water. Then, the majority of the THF was evaporated under vacuum. The resulting mixture was extracted three times with methylene chloride and washed with brine, and then water. The extract was dried over $MgSO_4$ and then the solvent evaporated under vacuum to yield the product as a pale, yellow oil. The crude product was purified by silica gel column chromatography (100% ethyl acetate) to yield 13.8 g (69% yield) of a clear viscous liquid (**4b**).

4a characterization: ^{1}H NMR (400 MHz, $CDCl_3$): 3.88-3.82 (m, 1H), 3.72-3.58 (m, 2H), 3.53-3.40 (m, 4H), 2.76 (s, 2H), 1.61-1.52 (m, 2H), 1.36-1.19 (m, 10H), 0.91-0.82 (t, 3H). ^{13}C NMR (125 MHz, $CDCl_3$) δ 72.4, 71.9, 70.8, 64.2, 31.9, 29.6, 29.5, 29.3, 26.2, 22.7, 14.2. HR-MS (ESI) calc for $C_{11}H_{24}O_3$ $[M+H]^+$ 227.16, found 227.10. FT-IR (ATR, ν, cm^{-1}) 3415 (m, broad) 2931 (m), 2859 (m), 1469 (w), 1381 (w), 1107 (s), 1052 (s), 958 (w), 869 (w), 758 (w), 751 (w)

4b characterization: ^{1}H NMR (500 MHz, $CDCl_3$): δ 5.84-5.68 (m, 1H), 5.00-4.84 (m, 2H), 3.86-3.77 (m, 1H), 3.68-3.50 (m, 2H), 3.46-3.35 (m, 6H), 2.06-1.93 (m, 2H), 1.62-1.45 (m, 2H), 1.41-1.18 (m, 6H) ^{13}C NMR (125 MHz, $CDCl_3$) δ 139.1, 114.4, 72.4, 71.8, 70.8, 64.2, 33.8, 29.6, 29.0, 28.9, 26.0. HR-MS (ESI) calc for $C_{11}H_{22}O_3$ $[M+H]^+$ 203.1642, found 203.1638. FT-IR (ATR, ν, cm^{-1}) 3410 (m, broad), 3088 (w), 2932 (m), 2861 (m), 1745 (w), 1643 (w), 1467 (w), 1246 (w), 1107 (s), 1049 (s), 910 (s), 873 (w), 732 (w)

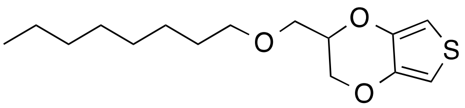

Compound **6a**

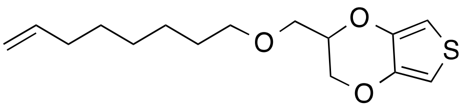

Compound **6b**

Figure S3. Compounds **6a** and **6b**

2-((octyloxy)methyl)-2,3-dihydrothieno[3,4-*b*][1,4]dioxine, **6a**, and 2-((oct-7-en-1-yloxy)methyl)-2,3-dihydrothieno[3,4-*b*][1,4]dioxine **6b**, were prepared in the same manner. A typical preparation for **6b** follows. To a flame-dried 200 mL round bottom flask equipped with a magnetic stir bar and a reflux condenser, p-tolunenesulfonic acid monohydrate (PTSA) (338 mg, 1.96 mmol), 40 mL of toluene was added. While stirring under Ar atmosphere, **4b** (5.96 g, 29.5 mmol) and 3,4-dimethoxythiophene (2.83 g, 19.6 mmol) (**5**) was added. 30 uL of water was added. The mixture was refluxed for 72 h under Ar atmosphere. After cooling, 40 mL of de-ionized water was added to the mixture to separate phases. The mixture was separated extracted three times with ethyl acetate, dried with $MgSO_4$ and evaporated under reduced pressure. The crude black-green oil was purified by silica gel column chromatography (10% ethyl acetate in hexanes). The purified product still contains up to 10% contamination of starting material (**5**). Vacuum distillation via Kugelrohr was used to yield 3.26 g (58.7%) of **6b**.

6a characterization: ^{1}H NMR (400 MHz, $CDCl_3$): 6.36-6.29 (q, 2H), 4.33-4.27 (m, 1H), 4.27-4.22 (m, 1H), 4.09-4.01 (m, 1H), 3.71-3.56 (m, 2H), 3.52-3.44 (m, 2H), 1.63-1.53 (m, 2H), 1.38-1.20 (m, 10H), 0.96-0.82 (m, 3H). ^{13}C NMR (125 MHz, $CDCl_3$) δ 139.8, 85.5, 73.3, 72.3, 68.6, 66.7, 31.9, 29.7, 29.5, 29.4, 26.2, 22.8, 14.3 HR-MS (ESI) calc for $C_{11}H_{24}O_3S$ $[M+H]^+$ 284.41, found 285.1971. FT-IR (ATR, ν, cm^{-1}) 3123 (m), 2927 (m), 2859 (m), 1586 (w), 1484 (s), 1454 (m), 1377 (m), 1185 (m), 1127 (m), 1022 (m), 949 (w), 939 (w), 859 (m), 754 (s)

6b characterization: ^{1}H NMR (500 MHz, $CDCl_3$): δ 6.40-6.25 (m, 2 H), 5.89-5.71 (m, 1H), 5.05-4.88 (m, 2H), 4.35-4.19 (m, 2H), 4.11-3.99 (m, 1H), 3.74-3.55 (m, 2H), 3.54-3.43 (m, 2H), 2.11-1.99 (m, 2H), 1.66-1.52 (m, 2H), 1.46-1.23 (m, 6H). ^{13}C NMR (125 MHz, $CDCl_3$) δ 141.7, 139.2, 114.4. 99.8, 72.8, 72.2, 69.2, 66.4, 33.8, 29.6, 29.0, 28.9, 26.0. HR-MS (ESI) calc for $C_{15}H_{24}O_3S$ $[M+H]^+$ 283.162, found 283.1368. FT-IR (ATR, ν, cm^{-1}) 3122 (w), 3085 (w), 2931 (m), 2861 (m), 1756 (w), 1642 (w), 1585 (w), 1485 (w), 1428 (m), 1376 (m), 1313 (w), 1185 (s), 1127 (s), 1023 (s), 912 (m), 860 (m), 755 (s), 733 (w)

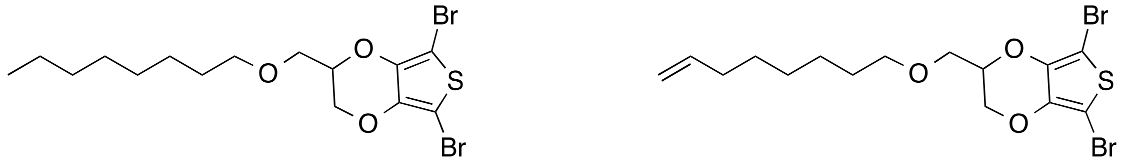

Compound **7a** Compound **7b**

Figure S4. Compounds **7a** and **7b**

5,7-dibromo-2-((octyloxy)methyl)-2,3-dihydrothieno[3,4-*b*][1,4]dioxine, **7a**, and 5,7-dibromo-2-((oct-7-en-1-yloxy)methyl)-2,3-dihydrothieno[3,4-*b*][1,4]dioxine **7b**, were prepared in the same manner. A typical preparation for **7b** follows. A flame dried 100 mL Schlenck flask was flushed with Ar (g) and charged with **6b** (6.44 g, 22.8 mmol) and dry THF (100 mL). The reaction mixture was cooled down to 0 °C, and N-bromosuccinimide (NBS) (8.12 g, 45.6 mmol) was added in four aliquots over the course of a half an hour. The reaction mixture was allowed to reach room temperature and stirred overnight. Then, it was added to 100 mL of water and extracted three times with ethyl acetate (100 mL). The combined organic layer was washed with brine and then dried over anhydrous $MgSO_4$, filtered, and concentrated under vacuum. The crude compound was purified by silica gel column chromatography on silica gel using 33% methylene chloride in hexanes as an eluent. The compound was dried under vacuum to yield 91.3 g (90.9%) of a viscous yellow oil, **7b**.

7a characterization: ^{1}H NMR (400 MHz, $CDCl_3$): 4.37-4.27 (m, 2H), 4.16-4.06 (m, 1H), 3.77-3.57 (m, 2H), 3.53-3.43 (m, 2H), 1.66-1.48 (m, 2H), 1.38-1.17 (m, 10H), 0.95-0.80 (t, 3H). ^{13}C NMR (125 MHz, $CDCl_3$) δ 139.7, 85.5, 72.3, 72.3, 68.6, 66.7, 31.9, 29.6, 29.5, 29.4, 26.2, 22.8, 14.3. -MS (ESI) calc for $C_{15}H_{22}$ Br_2 O_3S $[M+H]^+$ 442.97, found 440.97. FT-IR (ATR, ν, cm^{-1}) 2928 (m), 2858 (m), 1605 (w), 1507 (s), 1490 (w), 1415 (s), 1365 (s), 1221 (w), 1056 (s), 1017 (w), 965 (w), 916 (w), 916 (m), 759 (m), 669 (w)

7b characterization: ^{1}H NMR (500 MHz, $CDCl_3$): δ 5.86-5.74 (m, 1H), 5.02-4.89 (m, 2H), 4.38-4.27 (m, 2H), 4.18-4.06 (m, 1H), 3.78-3.57 (m, 2H), 3.53-3.43 (m, 2H), 2.09-1.97 (m, 2H), 1.64-1.51 (m, 2H), 1.45-1.24 (m, 6H). ^{13}C NMR (125 MHz, $CDCl_3$) δ 139.7, 139.1, 114.4, 85.5, 73.2, 72.2, 68.6, 66.7, 33.8, 29.6, 29.0, 28.9, 26.0. HR-MS (ESI) calc for $C_{15}H_{20}$ Br_2 O_3S $[M+H]^+$ 440.96, found 440.95. FT-IR (ATR, ν, cm^{-1}) 3085 (w), 2931 (m), 2860 (m), 1642, (w), 1605 (w), 1507 (s), 1415 (s), 1366 (s), 1335 (w), 1056 (s), 996 (m), 984 (m), 914 (s), 765 (m).

3. **Synthesis of polymers**

A. Kumada catalyst polycondensation polymerization.

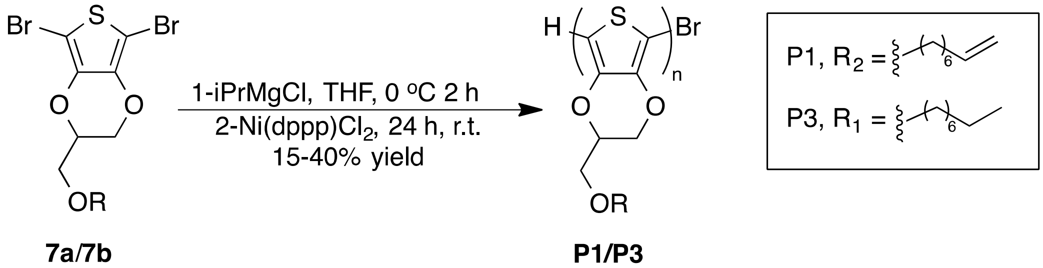

7a/7b **P1/P3**

Figure S5. Synthesis of **P1** and **P3**

P1 and P3 were prepared in the same manner. A typical preparation for **P1** follows. A solution of **7b** (1.00 g, 2.27 mmol) and 15 mL of dry, degassed THF in a 50 mL Schlenck tube was cooled to 0 ºC. Then, *i*PrMgCl•LiCl (329.9 mg, 2.27 mmol) was delivered dropwise via syringe. After 2 h, the reaction mixture was transferred via cannula to another 25 mL Schlenck tube containing 5 mL dry, degassed THF and Ni(dppp)Cl_2 (12.31 mg, 22.72 μmol) and allowed to react at room temperature for 24 h. The reaction was quenched with 0.50 mL of degassed 6.0 M $HCl_{(aq)}$. The crude mixture was precipitated in 200 mL cold methanol containing 5.0 mL hydrazine hydrate. The resulting suspension of polymer was filtered to afford a black solid. This solid was placed in a Soxhlet thimble and extracted

with methanol, hexanes and THF. The solvent was removed under vacuum and the remaining polymer was placed in a vacuum oven overnight to yield 441 mg (44%) of a purple and copper colored solid, **P1**.

P1 characterization: ^{1}H NMR (500 MHz, $CDCl_3$): 6.626 (m, 0.05 H, end group), 5.94-5.53 (m, 1H), 5.13-4.69 (m, 2H), 4.62-3.99 (m, 3H), 3.94-3.07 (m, 4H), 2.04 (m, 2H), 1.59 (m, 2H), 1.36 (m, 2H). FT-IR (ATR, ν, cm^{-1}) 2939 (broad, weak), 2872 (broad, weak), 1666 (broad, weak), 1519 (broad, weak), 1465 (sharp, medium), 14315 (sharp, medium), 1316 (broad, medium), 1208 (sharp, medium), 1107 (sharp, medium), 1045 (sharp, medium), 976 (sharp, medium),

P3 characterization: ^{1}H NMR (400 MHz, $CDCl_3$): δ 6.27 (0.06, m, end group) 4.47 (m, 2H), 4.22 (m, 1H), 3.94-3.69 (m, 2H), 3.57 (m, 2H), 1.57 (m, 2H), 1.38-1.27 (m, 10H), 0.87 (t, 3H). FT-IR (ATR, ν, cm^{-1}) 2927 (sharp, med), 2860 (sharp, med), 1518 (broad, weak), 1465 (sharp, med), 1435 (sharp, strong), 1380 (sharp, med), 1310 (sharp, med), 1200 (broad, med), 1107 (sharp, strong), 1079 (sharp, strong), 1043 (sharp, strong), 989 (broad, med), 723 (sharp, weak), 639 (sharp, medium)

B. Post-polymerization modification via alkene cross metathesis.

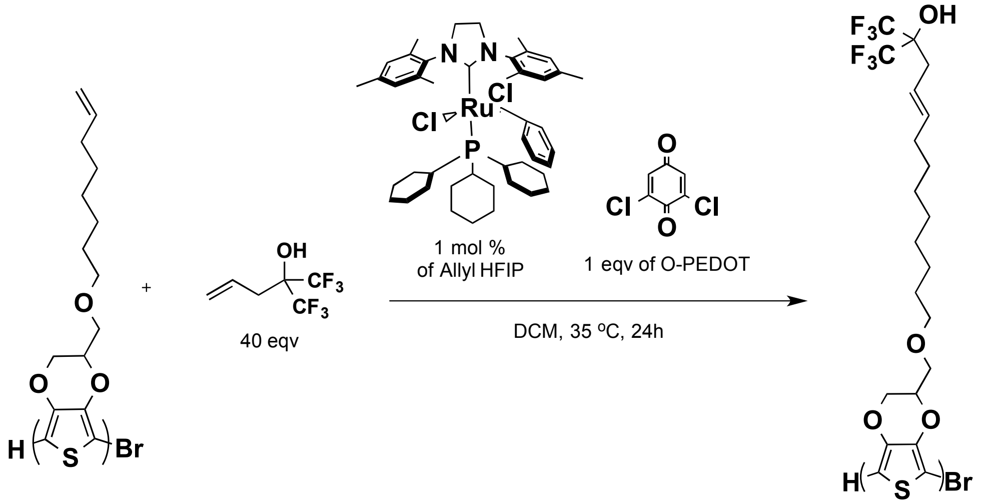

Figure S6. Synthesis of **P2**

To a 50 mL round bottom flask equipped with a magnetic stir bar and a reflux condenser, 20 mL degassed methylene chloride was added. Then, allyl-hexafluoroisopropanol (4.5 g, 21.6 mmol), 2,5-dichlorobenzoquinone (76.0 mg, 0.432 mmol) and **P1** (121 mg, 0.432 mmol) were delivered to the flask and sparged for 10 minutes with $Ar_{(g)}$. Grubbs' II catalyst (184 mg, 0.216 mmol) was added and the reaction was heated to 35 °C and was allowed to react for 24 hours. The reaction was quenched with the addition of 0.10 mL of ethyl vinyl ether and the mixture was precipitated in 175 ml cold acetonitrile containing 5.0 mL hydrazine hydrate. The resulting suspension of polymer was filtered to afford a black solid. This solid was placed in a Soxhlet thimble and extracted with methanol and hexanes. Then, the crude polymer was dissolved in THF and placed in a 2000 MWCO dialysis bag. The dissolved polymer in was placed in a beaker of stirring THF and allowed to remain for 24 hours. The THF was then evaporated and the solid was placed in a vacuum oven over night to yield 166 mg (78%) of a dark navy blue powdered solid, **P2**.

P2 characterization: ^{1}H NMR (500 MHz, THF-d_8): 6.31 (m, 0.16 H, end group), 5.88-5.35 (m, 2H), 4.49 (m, 2H) , 4.24 (m, 2H), 3.85, 2.67, 2.08, 1.91. FT-IR (ATR, ν, cm^{-1}) 3518-3121 (broad, weak), 2932 (broad, weak), 2861 (broad, weak), 1519 (sharp, med), 1344 (broad, strong) 1204 (sharp, strong), 1053 (broad, strong), 978 (sharp, med)

Figure **S7**: Gel Permeation Chromatogram (GPC) of **P1**-**P3** (THF fractions)

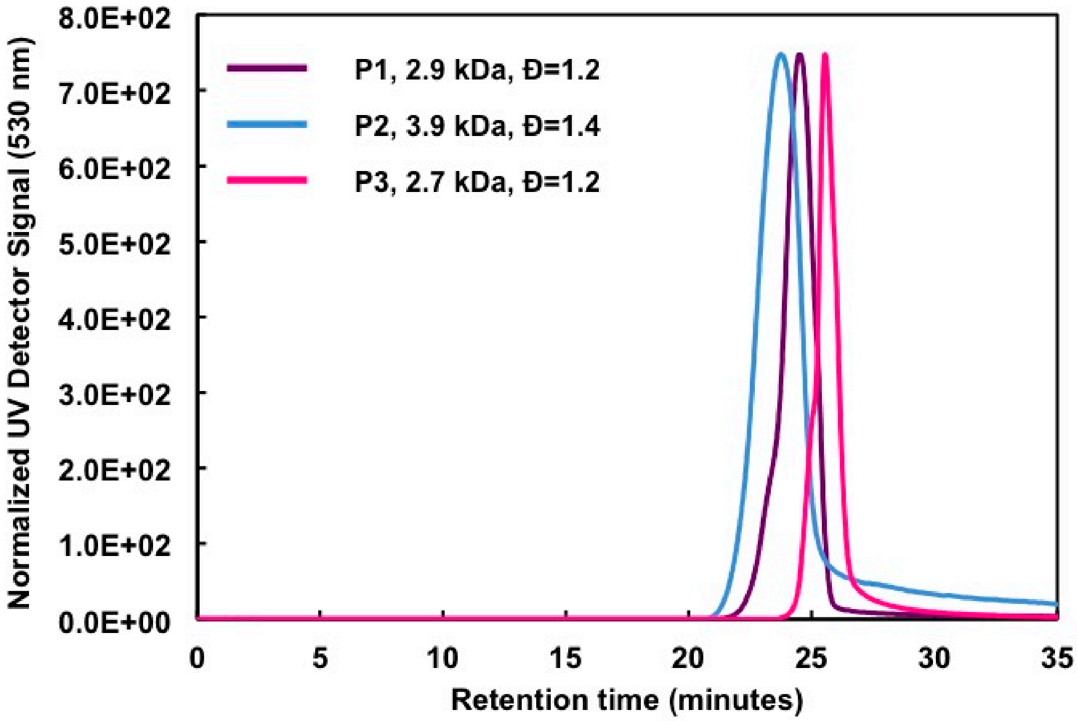

Table **S1**: Molecular Weight Analysis

**Analytical Technique**	**P1 MW (repeat units)**	**P2 MW (repeat units)**	**P3 MW (repeat units)**
GPC	2,900 g/mol<br>Đ=1.2<br>(10)	3,900 g/mol<br>Đ=1.2<br>(8)	2,700 g/mol<br>Đ=1.2<br>(9)
^{1}H NMR (end group analysis)	5,800 g/mol<br>(20)	6,600 g/mol<br>(13)	9,300 g/mol<br>(33)
MALDI-TOF MS	2,245 g/mol<br>(8)	1322 g/mol<br>(3)	3,395 g/mol<br>(12)

Figure **S8**: MALDI-TOF MS of Compound **P1**- poly(octenylEDOT), high molecular weight, sparingly soluble fraction

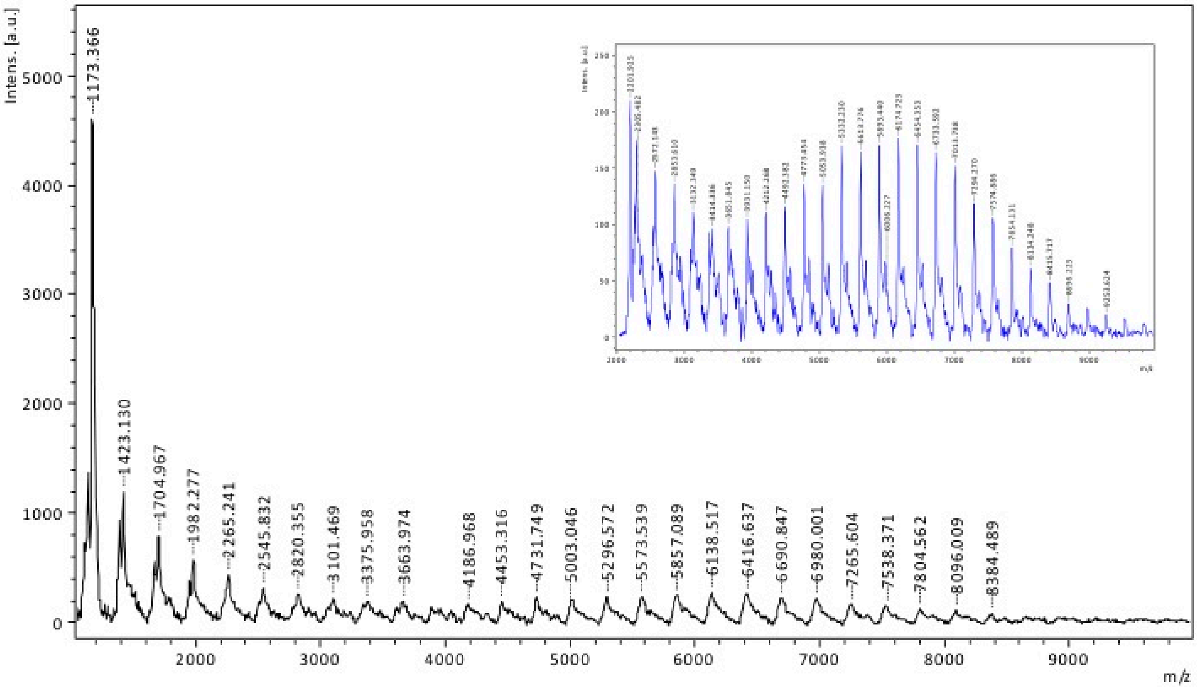

Figure **S9**: MALDI-TOF MS of Compound **P1**- poly(octenylEDOT)

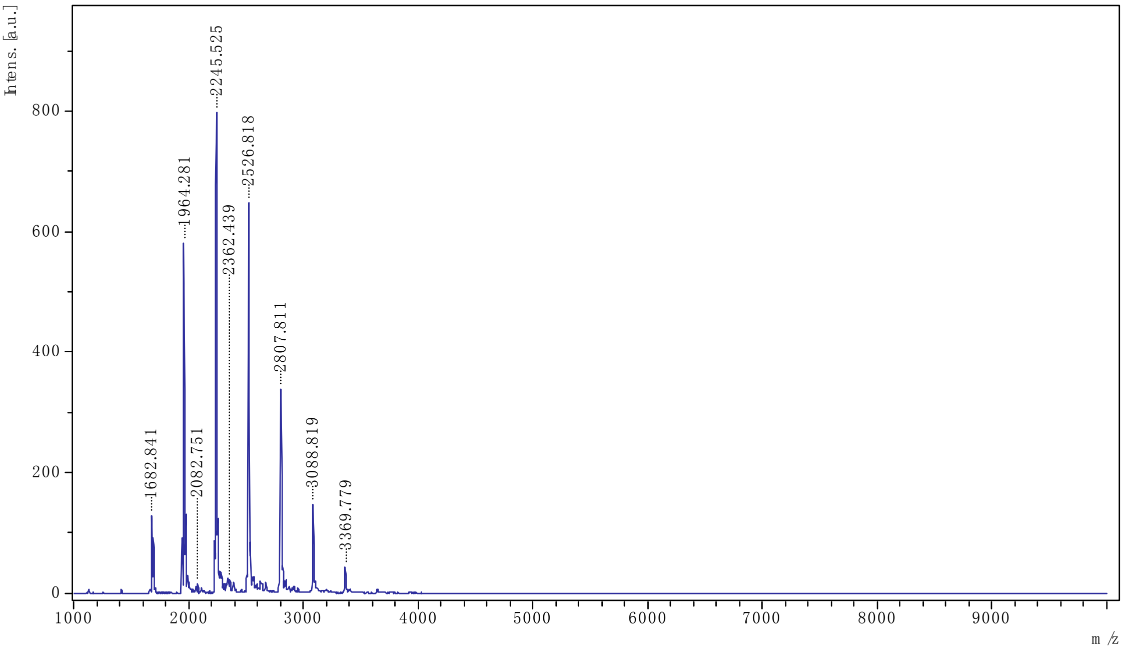

Figure **S10**: MALDI-TOF MS Compound **P2**- Poly(octenyl-HFIP-PEDOT)

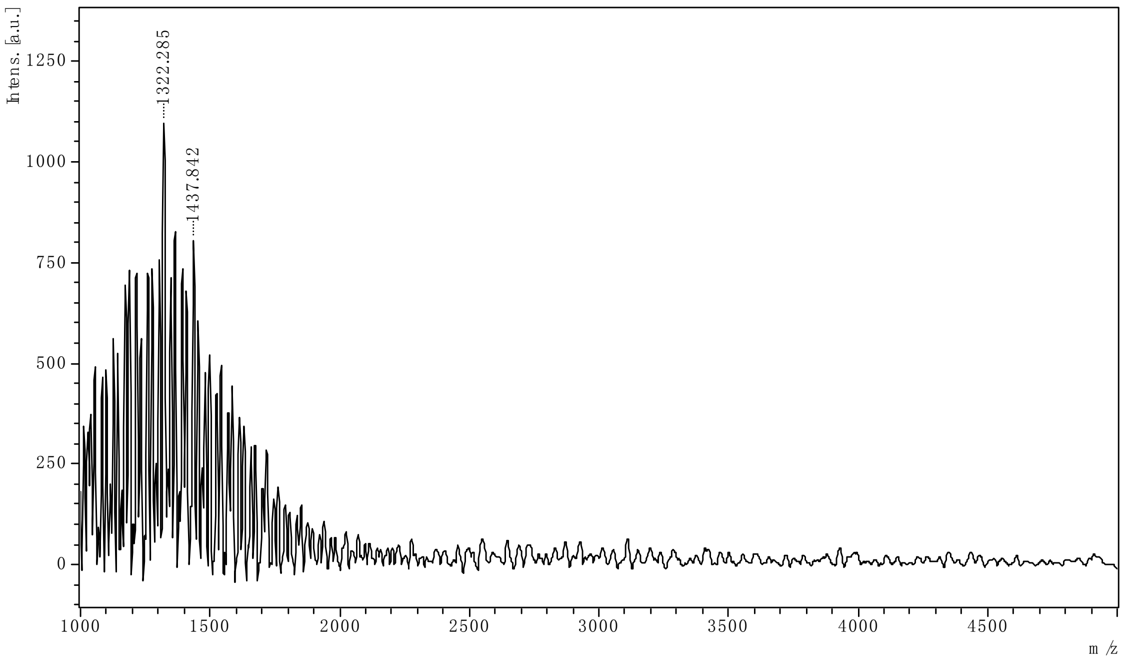

Figure **S11**: MALDI-TOF MS Compound **P3**- poly(octylPEDOT)

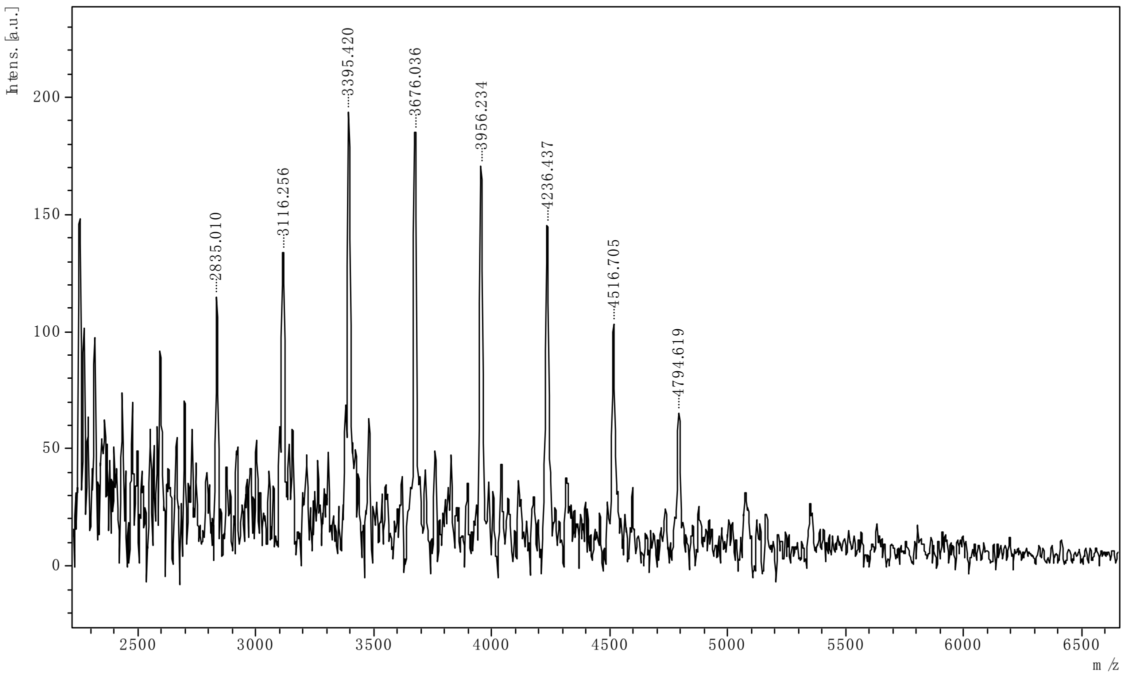

Figure **S12**: ^{1}H NMR of Compound **P1**- poly(octenylEDOT)

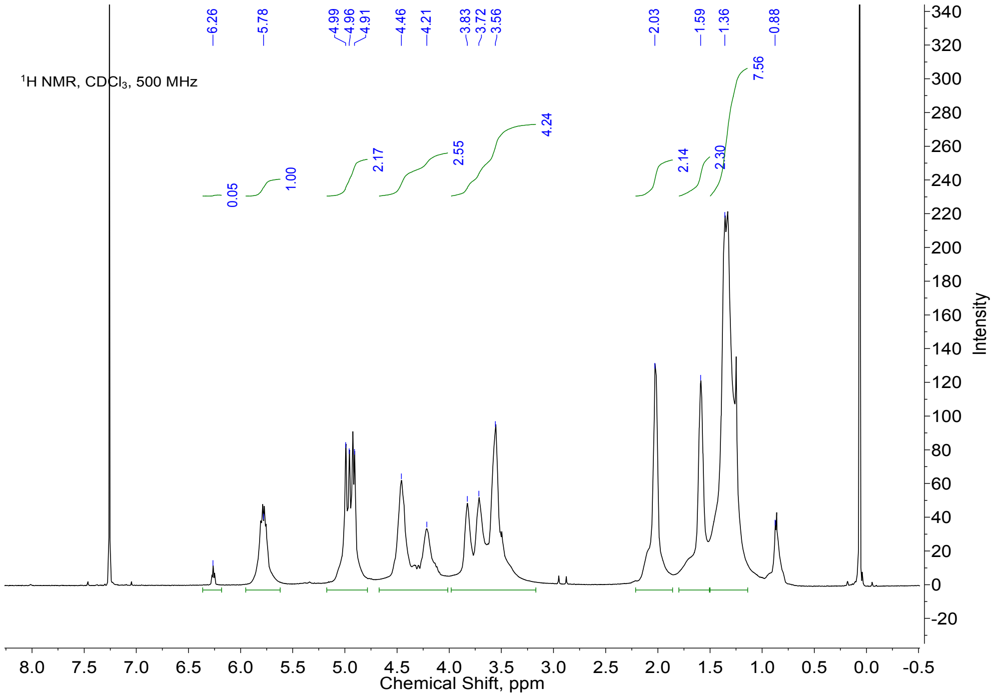

Figure **S13**: ^{1}H NMR of Compound **P2**- Poly(octenyl-HFIP-PEDOT)

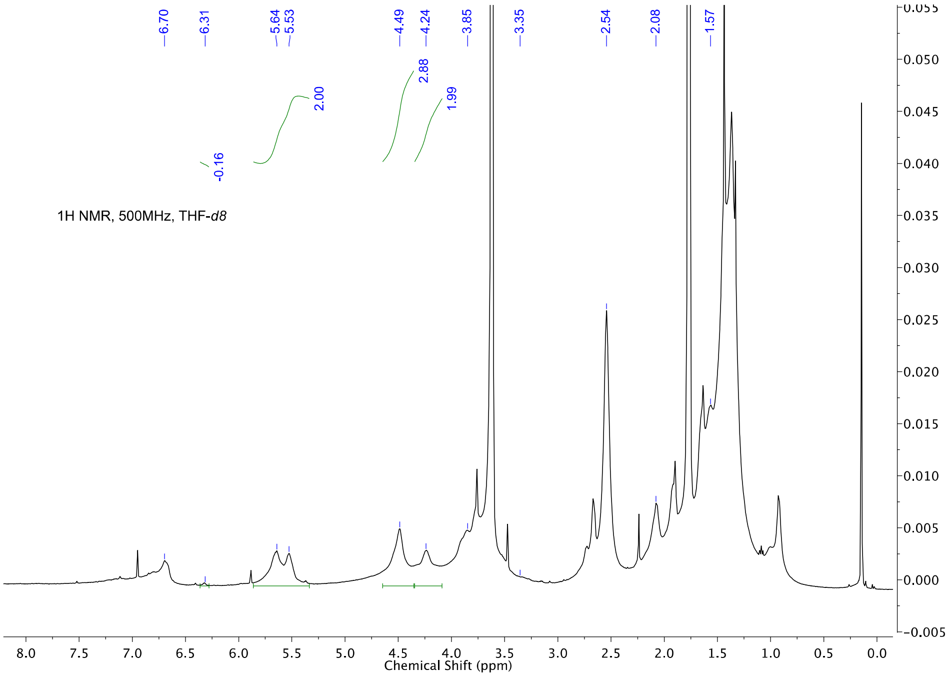

Figure **S14**: ^{19}F NMR of Compound **P2**- Poly(octenyl-HFIP-PEDOT)

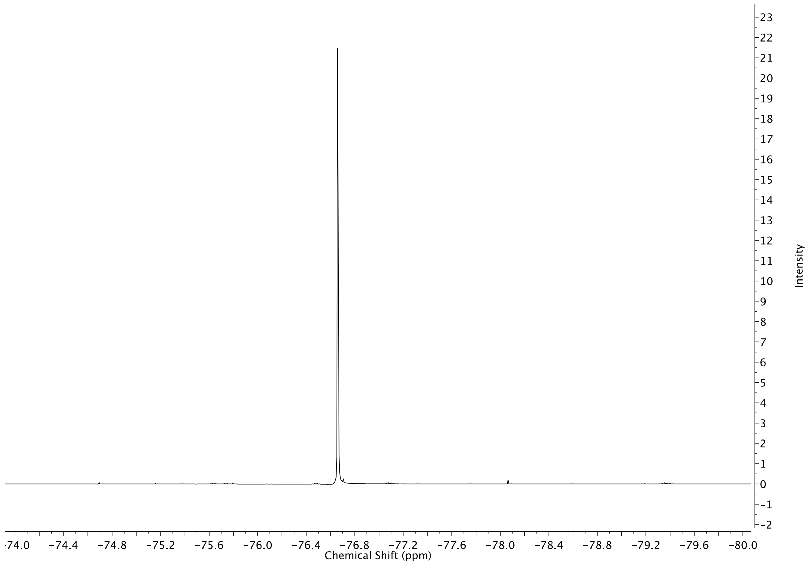

Figure **S15**: Compound **P3**- poly(octylEDOT)

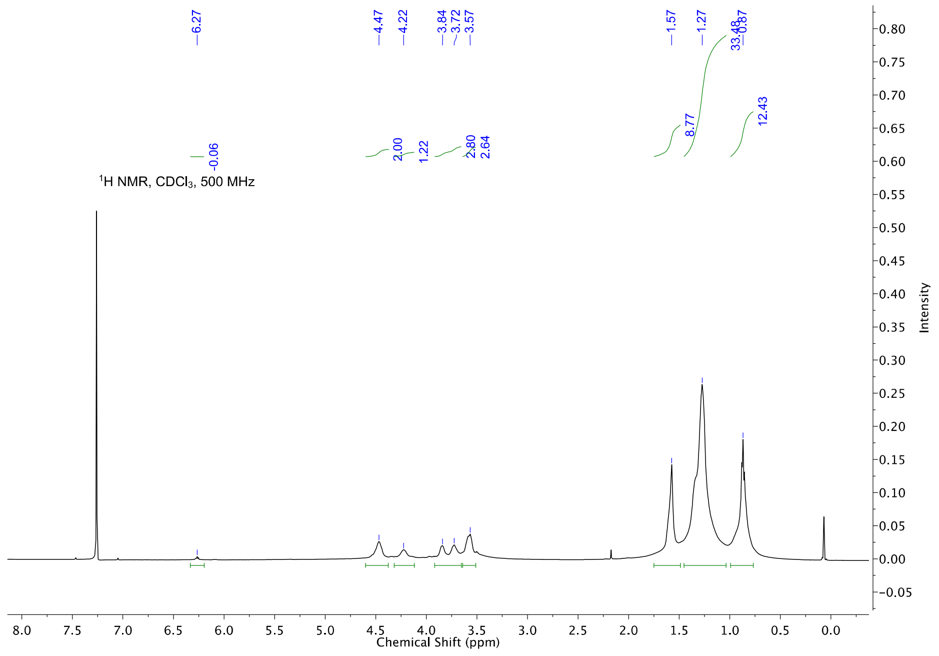

Figure **S16**: Response of three devices with three ratios of P2/SWCNT to 2.0 ppm DMMP for a 60 second exposure with N_2 as a diluent gas.

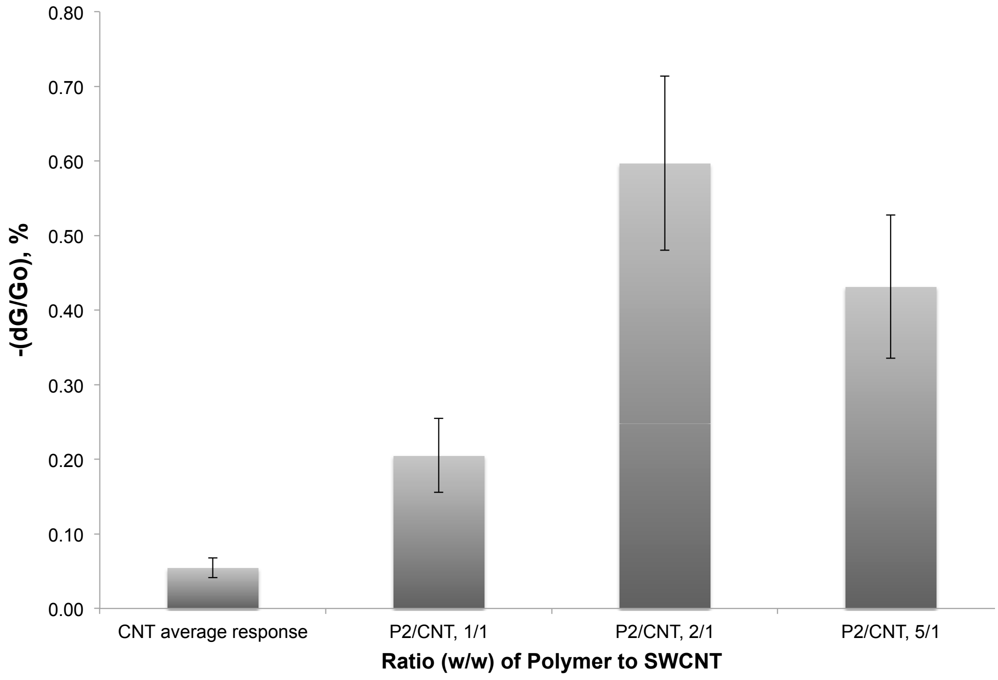

Figure **S17**: Full Raman Spectra of pristine SWCNT, P1/SWCNT, P2/SWCNT, P3/SWCNT taken at an excitation wavelength of 633 nm

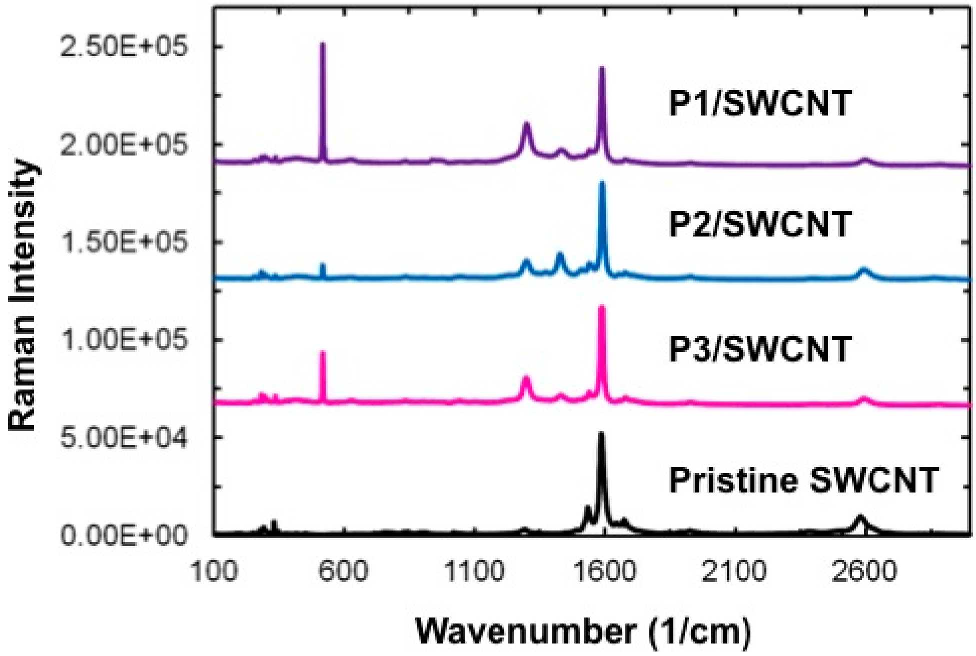

Figure **S18**: Full Raman Spectra of **P1-P3** and Pristine SWCNTs taken at an excitation wavelength of 633 nm

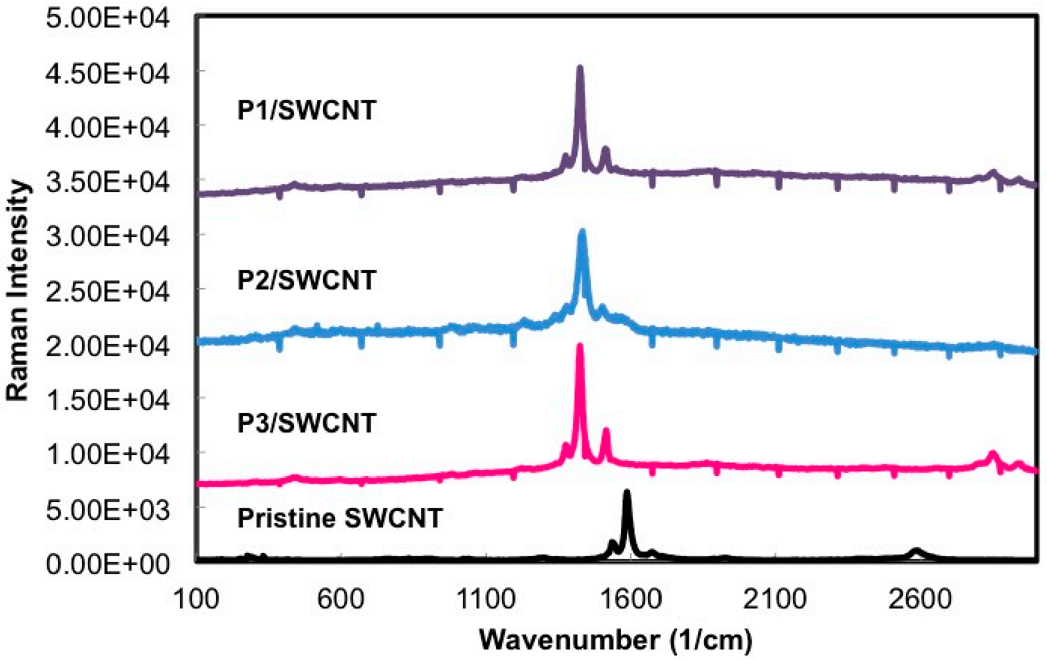

Figure **S19**: Combined sensing traces of a pristine SWCNT device (black trace) and P2/SWCNT (blue trace) and P3/SWCNT devices (pink trace) to 60 s exposures followed by of VOCs at a flow rate of 200 mL/min. The traces are offset for clarity.

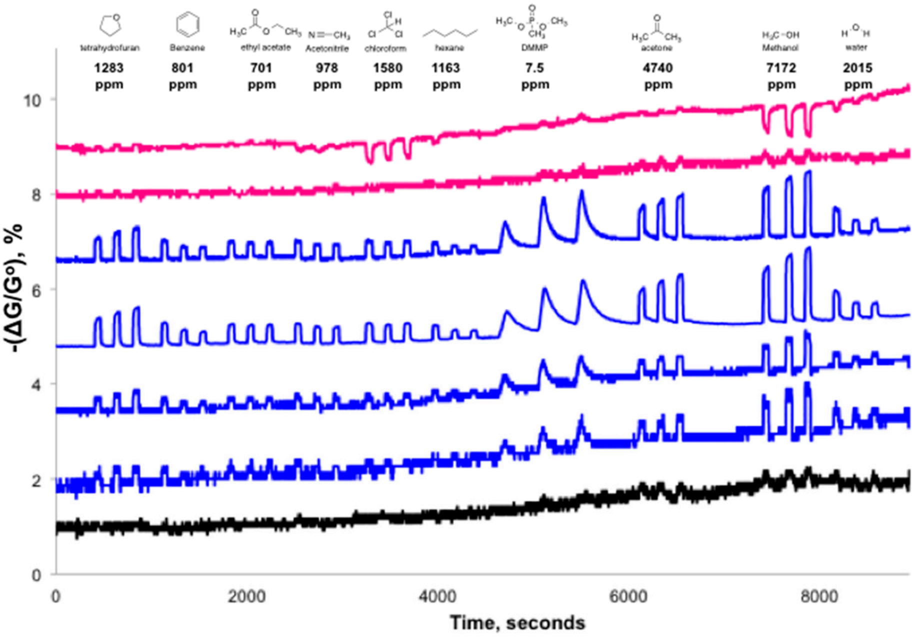

Figure **S19a:** Sensing trace of a pristine SWCNT device resulting from 60 s exposures followed by of VOCs at a flow rate of 200 mL/min.

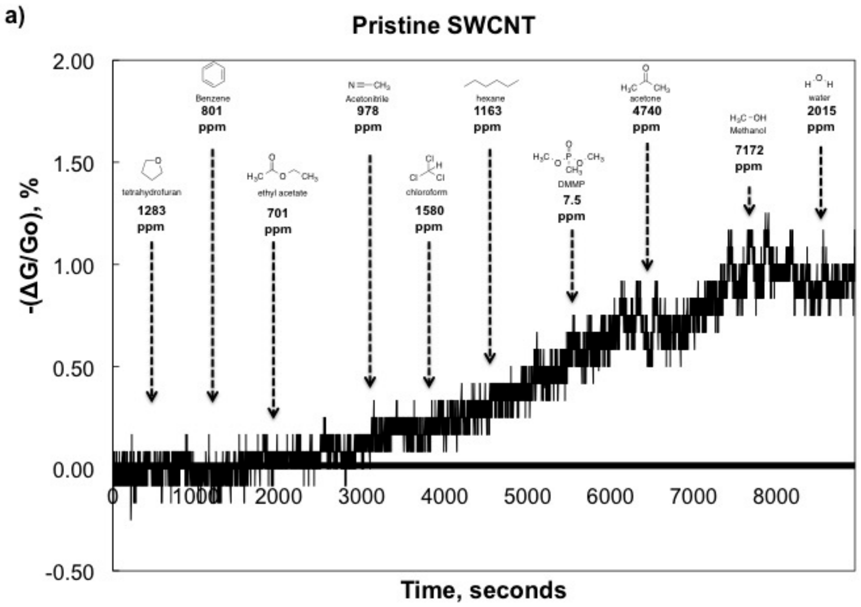

Figure **S19b:** Sensing trace of a P2/SWCNT device resulting from 60 s exposures followed by of VOCs at a flow rate of 200 mL/min.

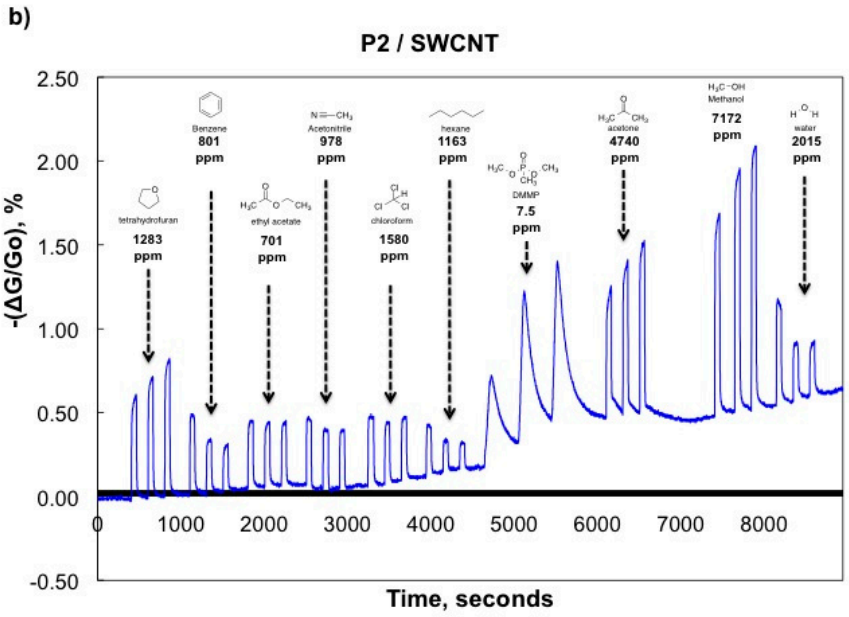

Figure **S19c:** Sensing trace of a P3 / SWCNT device resulting from 60 s exposures followed by of VOCs at a flow rate of 200 mL/min.

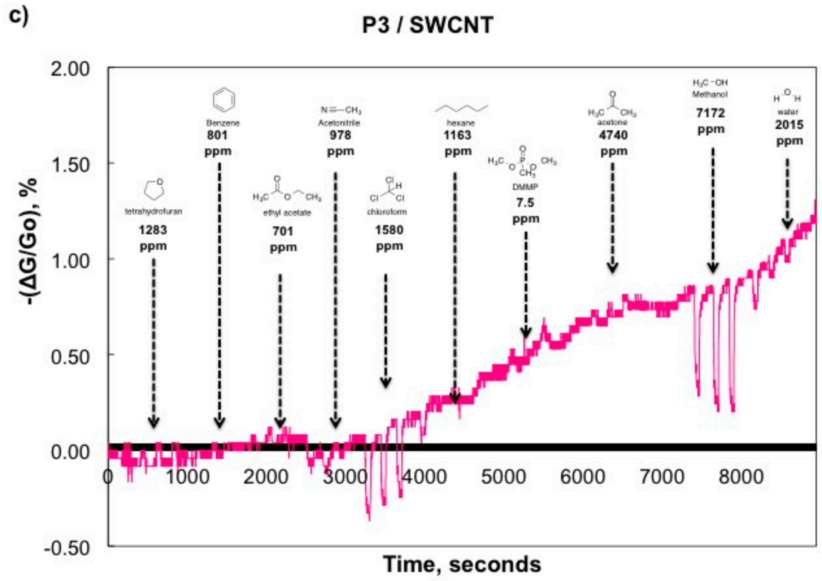

Figure **S20**: ^{1}H and ^{13}C NMR of Compound **3a -** 2,2-dimethyl-4-((octyloxy)methyl)-1,3-dioxolane

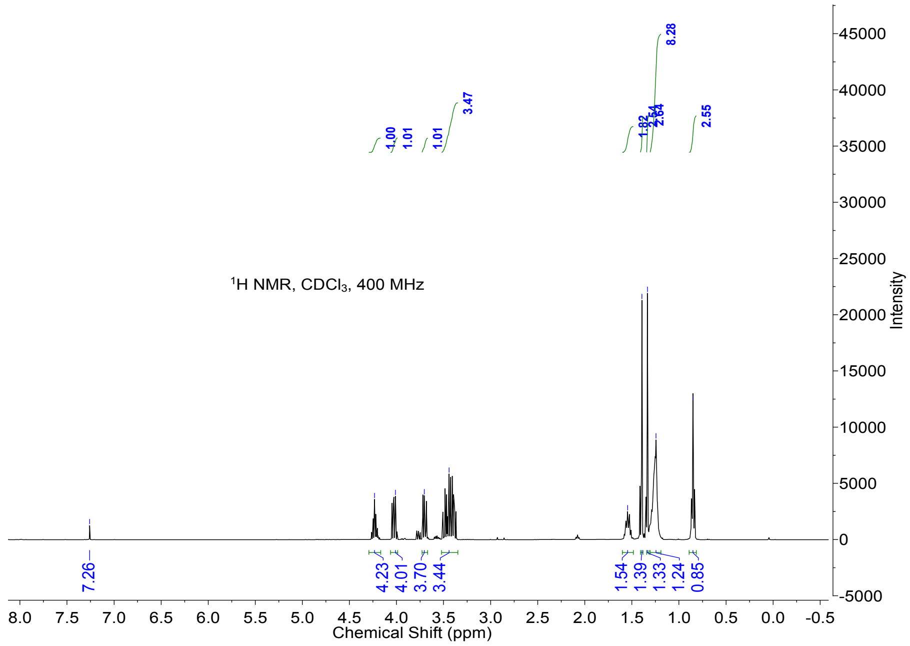

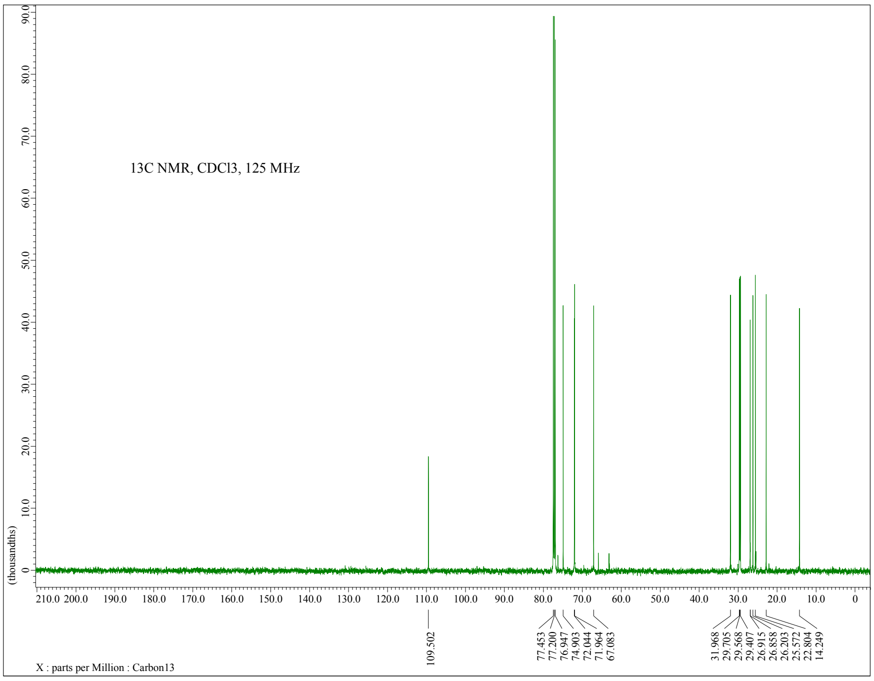
13C NMR, CDCl3, 125 MHz
(thousandths)
X : parts per Million : Carbon13
109.502
77.453
77.200
76.947
74.903
72.044
71.964
67.083
31.968
29.705
29.568
29.407
26.915
26.858
26.203
25.572
22.804
14.249

Figure **S21**: ^{1}H and ^{13}C NMR of Compound **3b** - 2,2-dimethyl-4-((oct-7-en-1-yloxy)methyl)-1,3-dioxolane

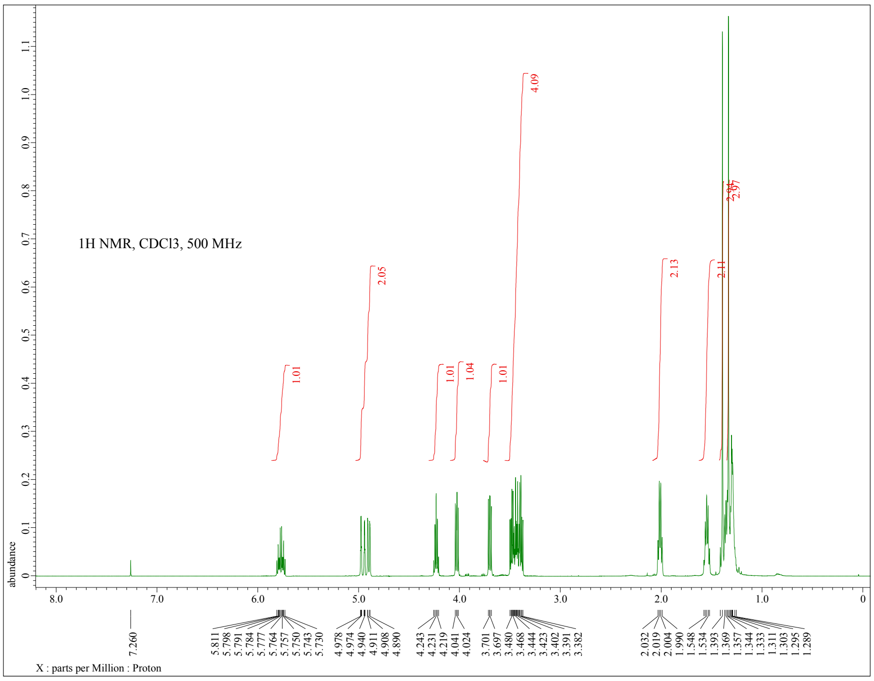

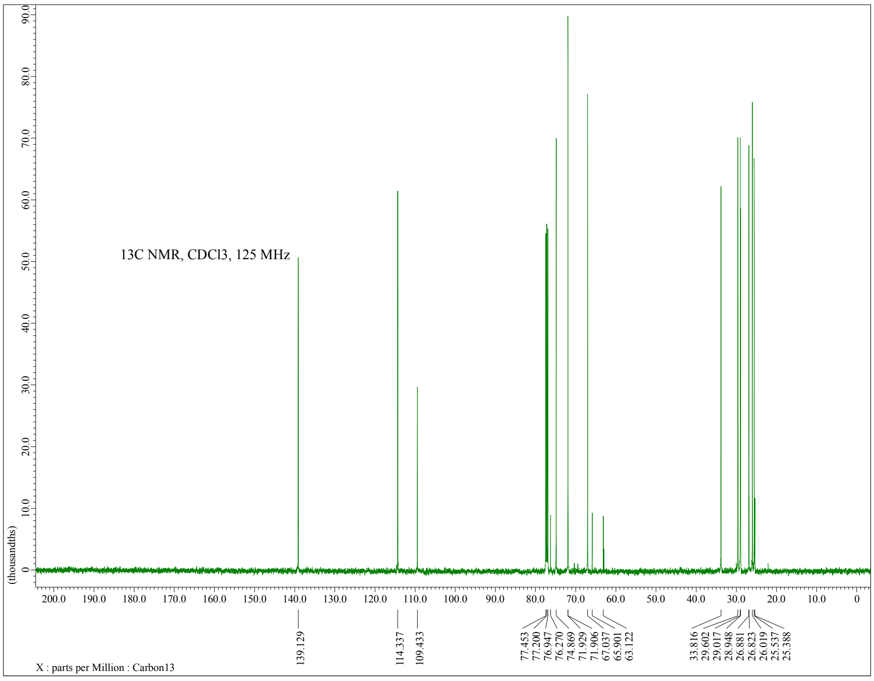

Figure **S22**: ^{1}H and ^{13}C NMR of Compound **4a** 3-(octyloxy)propane-1,2-diol

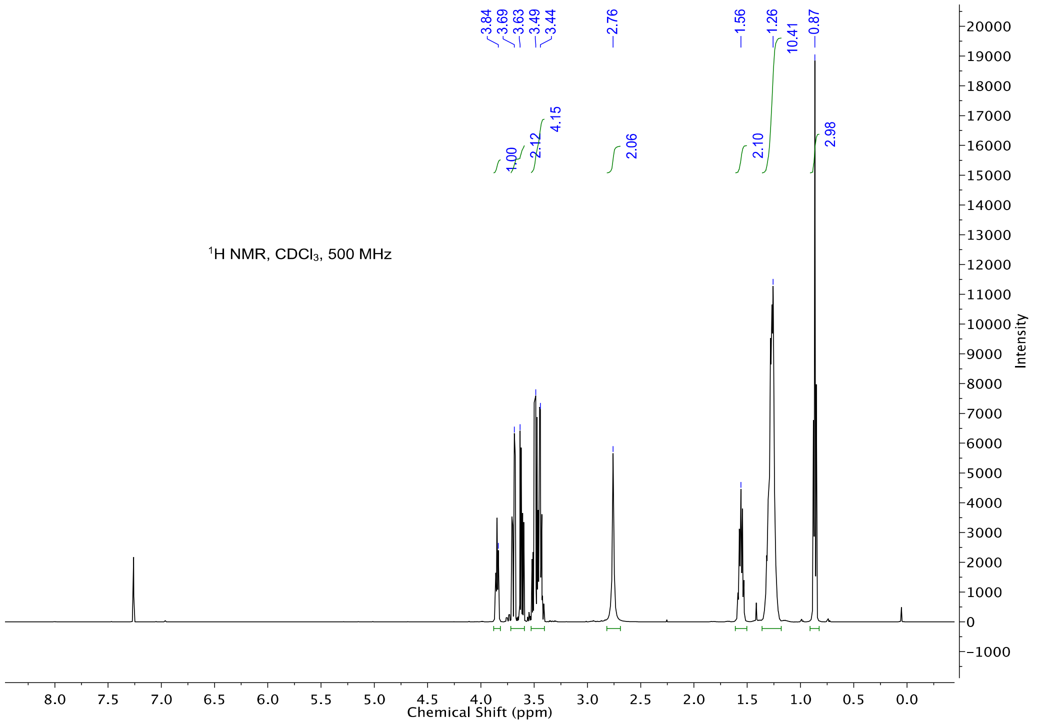

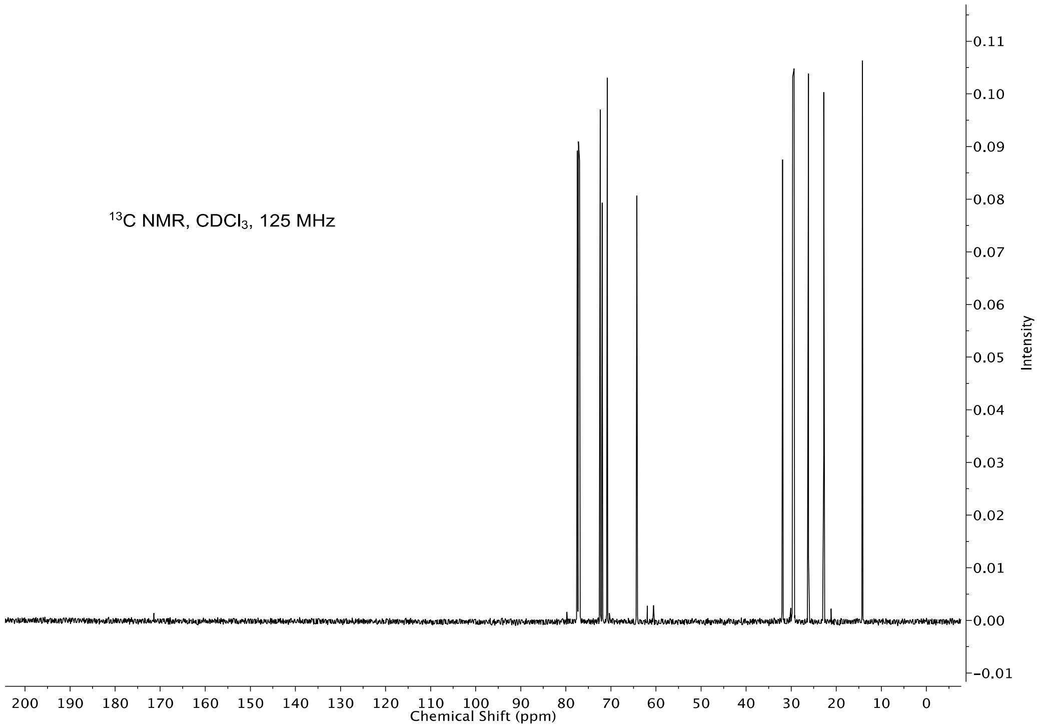

Figure **S23**: ^{1}H and ^{13}C NMR of Compound **4b** - 3-(oct-7-en-1-yloxy)propane-1,2-diol

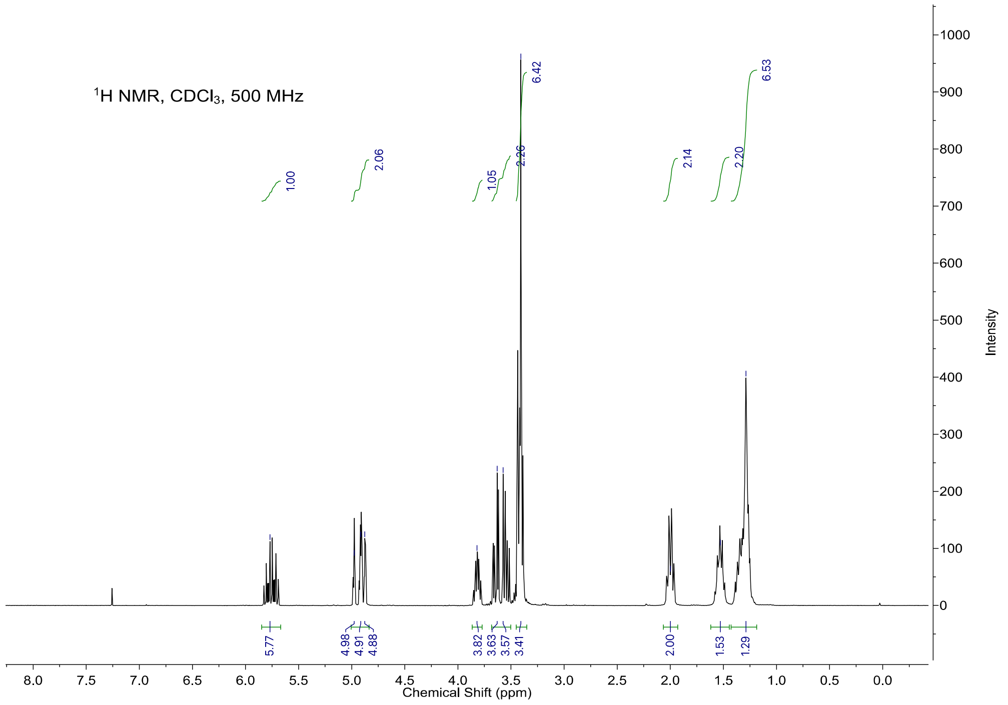

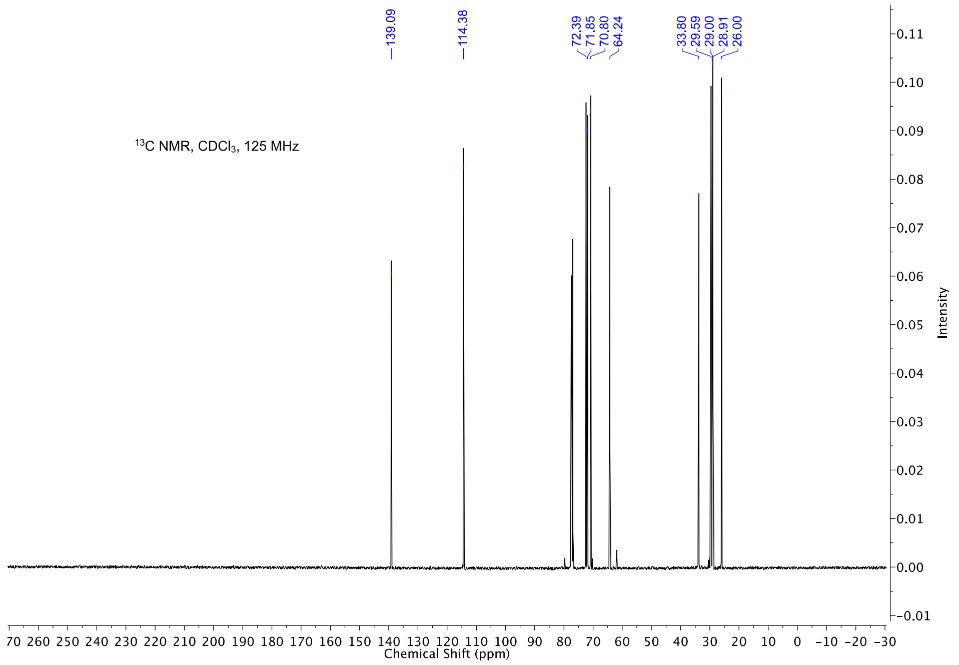

Figure **S24**: ^{1}H and ^{13}C NMR of Compound **6a** - 2-((octyloxy)methyl)-2,3-dihydrothieno[3,4-*b*][1,4]dioxine

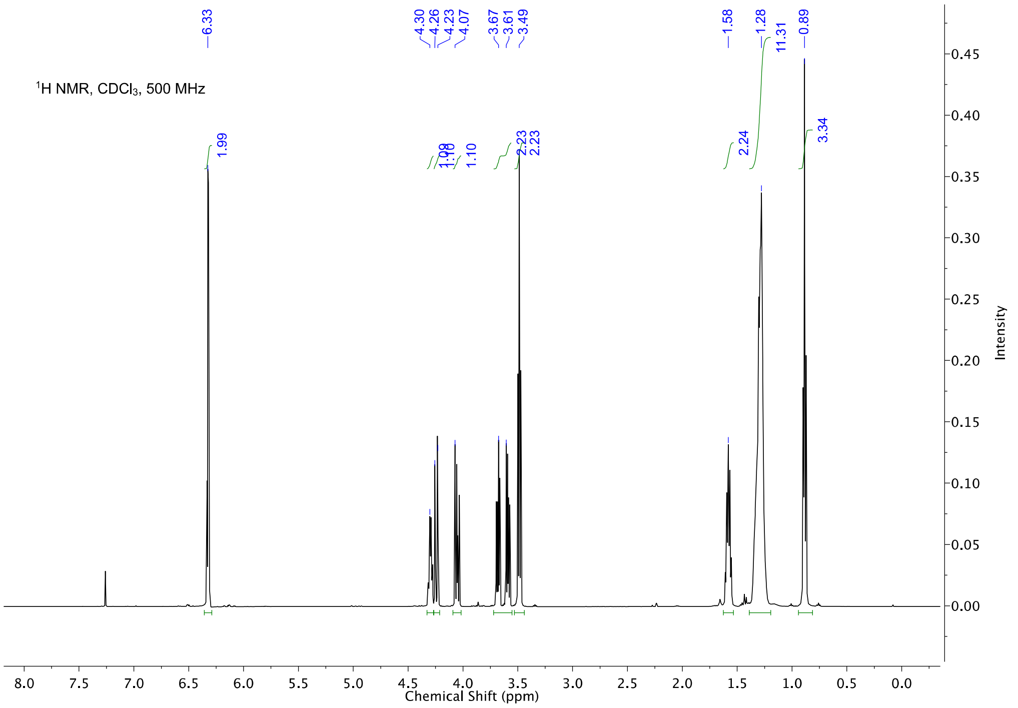

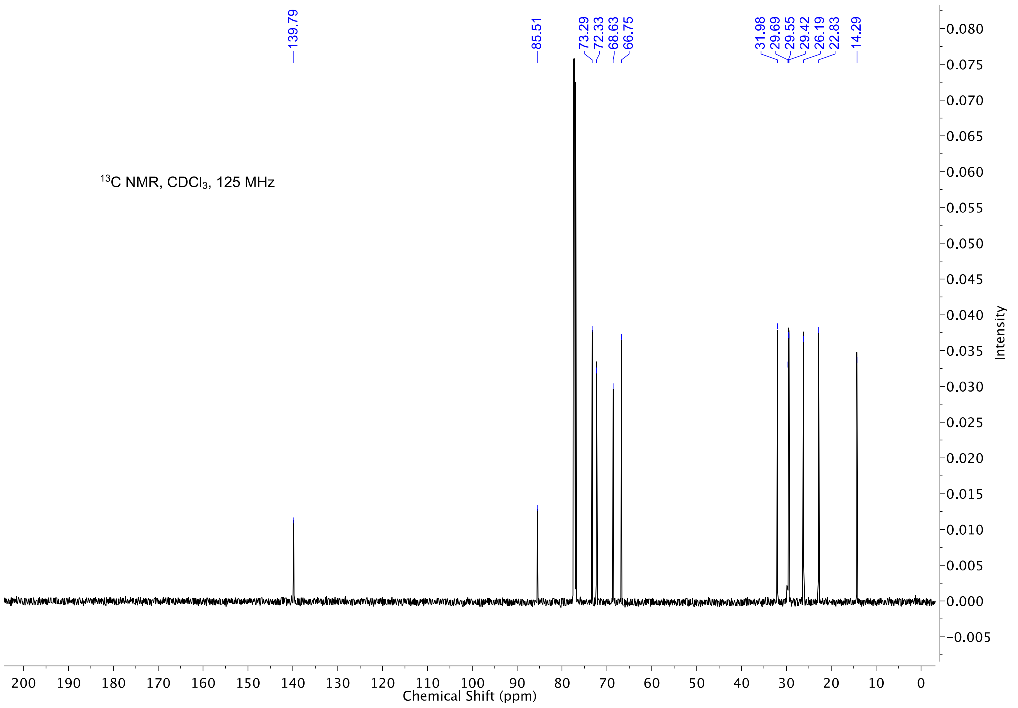

Figure **S25**: ^{1}H and ^{13}C NMR of Compound **6b** - 2-((oct-7-en-1-yloxy)methyl)-2,3-dihydrothieno[3,4-*b*][1,4]dioxine

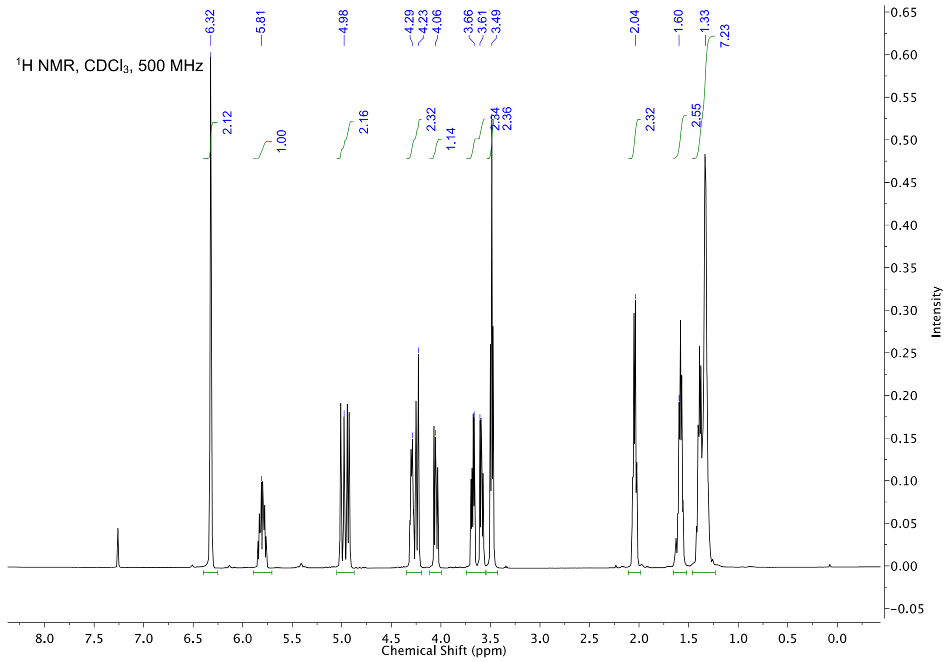

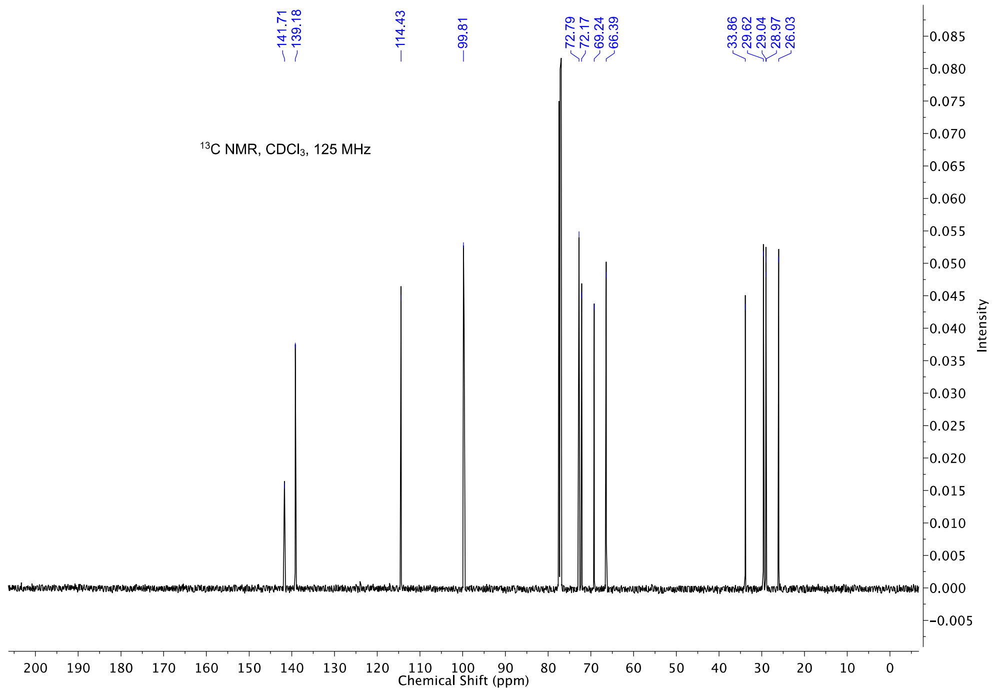

Figure **S26**: ^{1}H and ^{13}C NMR of Compound **7a** - 5,7-dibromo-2-((octyloxy)methyl)-2,3-dihydrothieno[3,4-*b*][1,4]dioxine

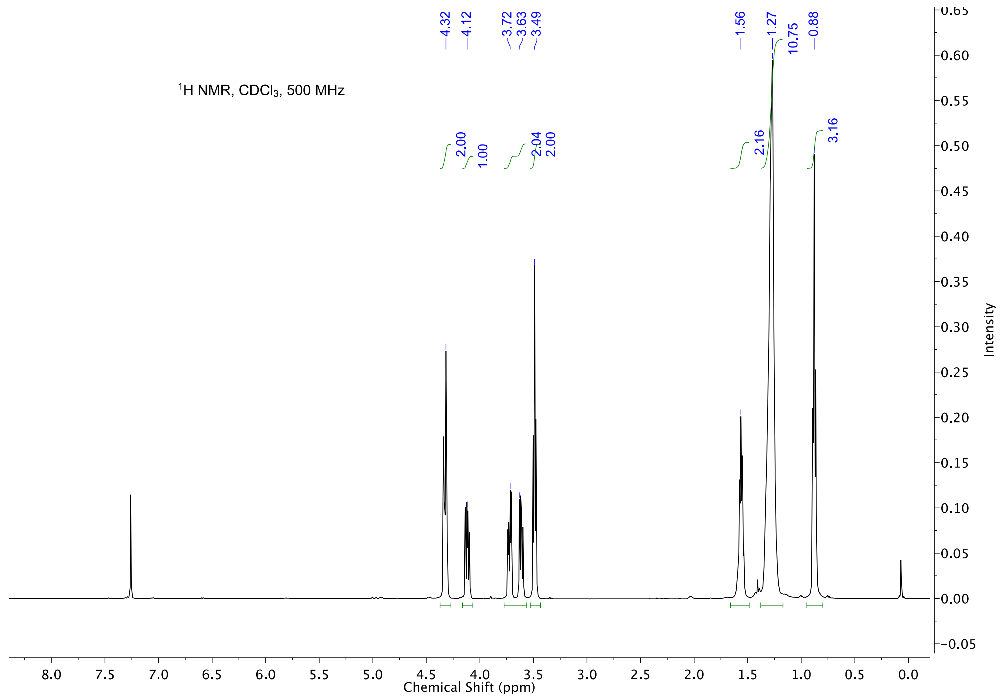

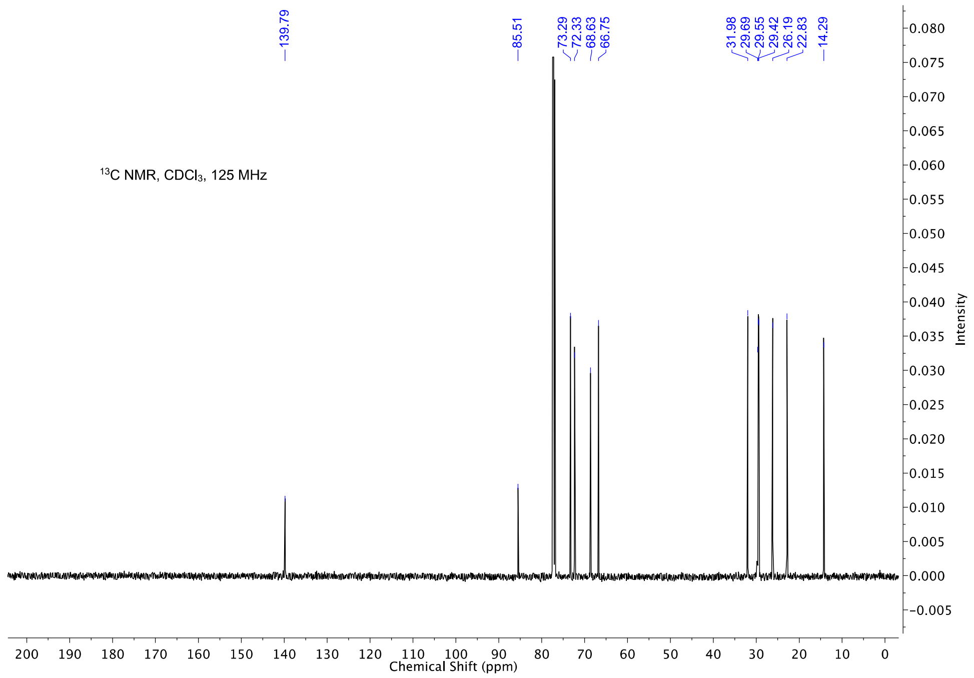

Figure **S27**: ^{1}H and ^{13}C NMR of Compound **7b** - 5,7-dibromo-2-((oct-7-en-1-yloxy)methyl)-2,3-dihydrothieno[3,4-*b*][1,4]dioxine

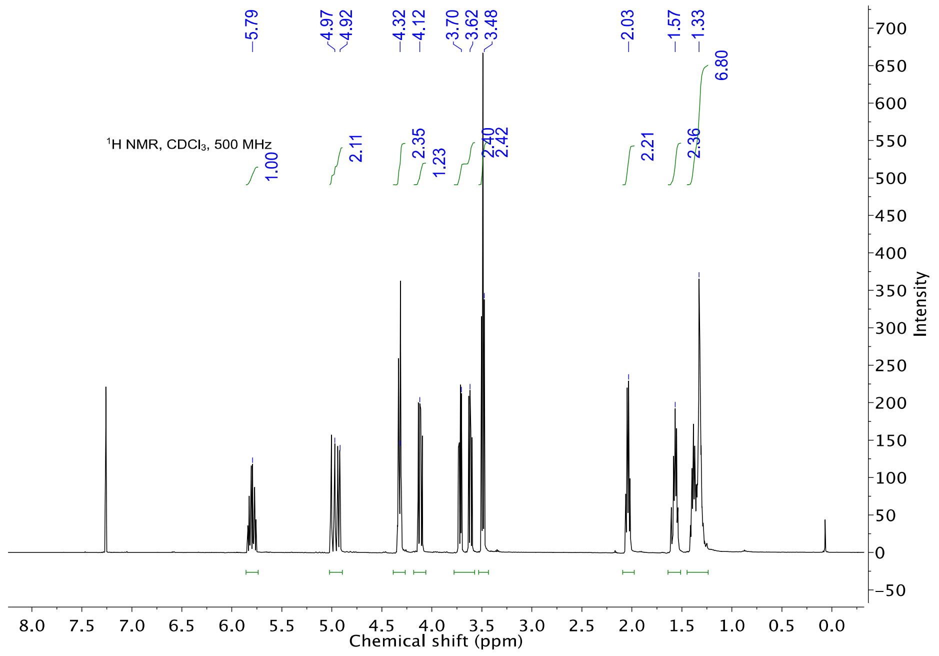

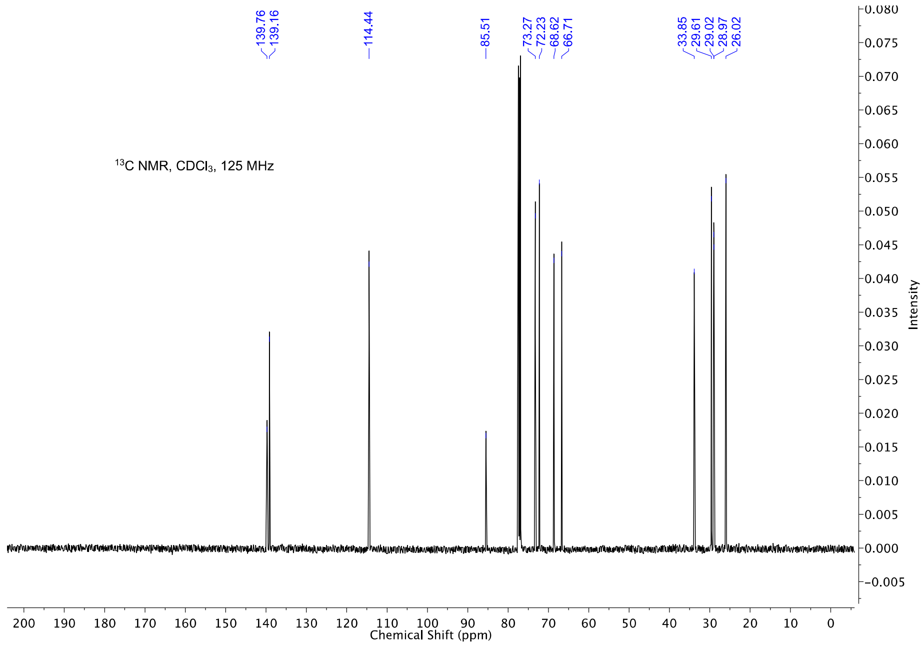

Figure **S28**: FTIR-ATR of Compound **3a -** 2,2-dimethyl-4-((octyloxy)methyl)-1,3-dioxolane

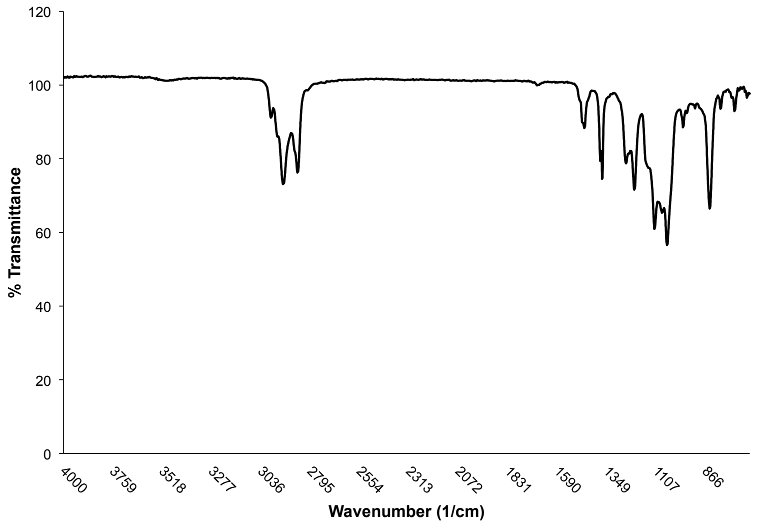

Figure **S29**: FTIR-ATR of Compound **3b** - 2,2-dimethyl-4-((oct-7-en-1-yloxy)methyl)-1,3-dioxolane

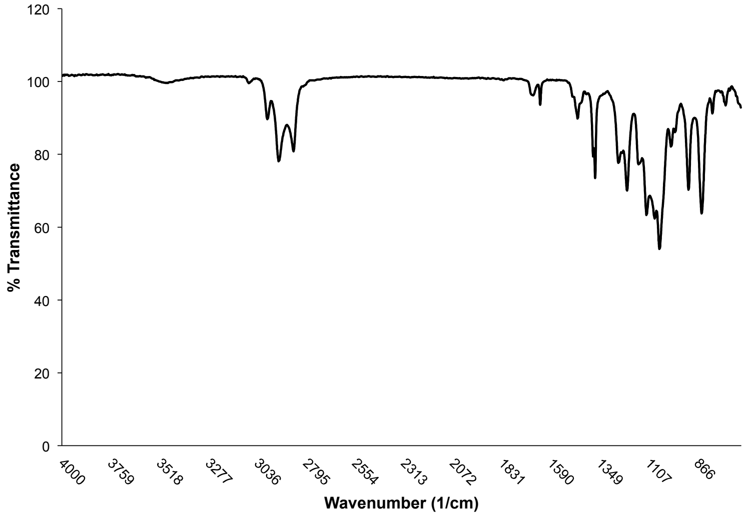

Figure **S30**: FTIR-ATR of Compound **4a** 3-(octyloxy)propane-1,2-diol

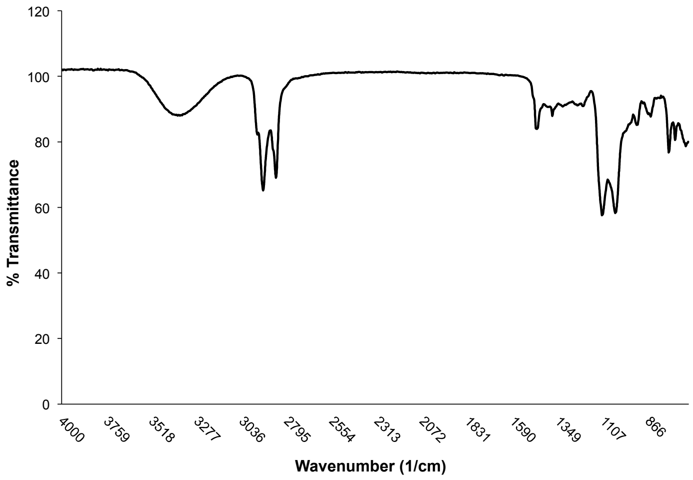

Figure **S31**: FTIR-ATR of Compound **4b** - 3-(oct-7-en-1-yloxy)propane-1,2-diol

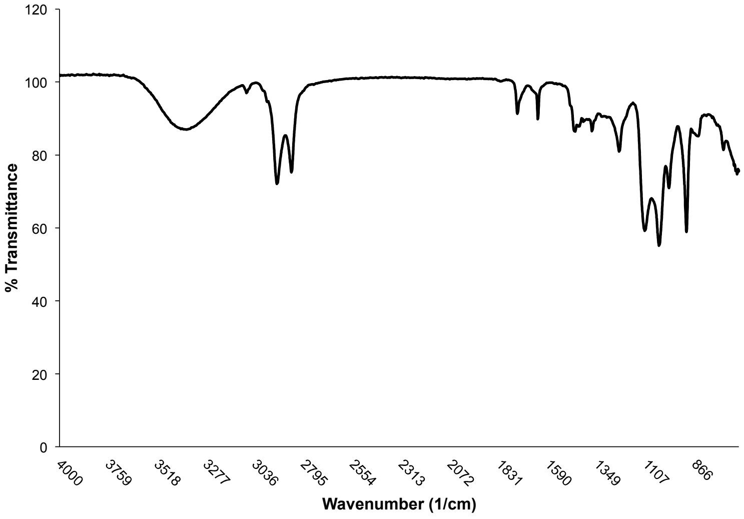

Figure **S32**: FTIR-ATR of Compound **6a** - 2-((octyloxy)methyl)-2,3-dihydrothieno[3,4-*b*][1,4]dioxine

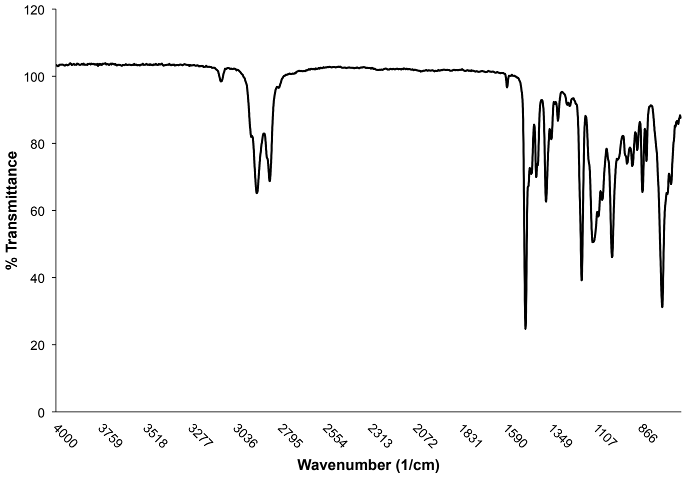

Figure **S33**: FTIR-ATR of Compound **6b** - 2-((oct-7-en-1-yloxy)methyl)-2,3-dihydrothieno[3,4-*b*][1,4]dioxine

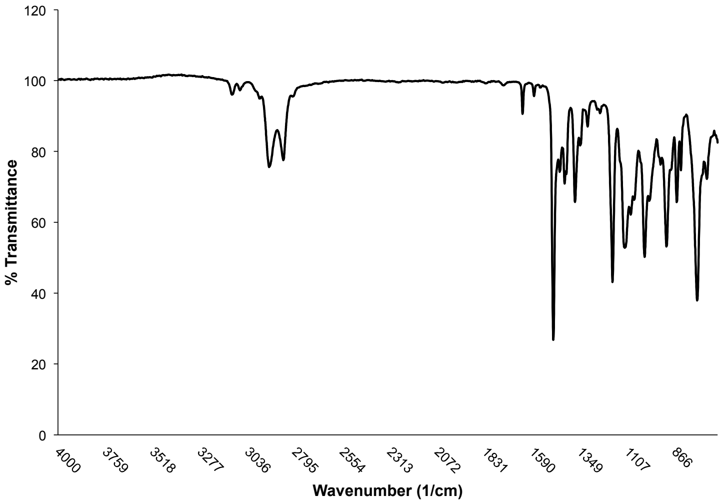

Figure **S34**: FTIR-ATR of Compound **7a** - 5,7-dibromo-2-((octyloxy)methyl)-2,3-dihydrothieno[3,4-*b*][1,4]dioxine

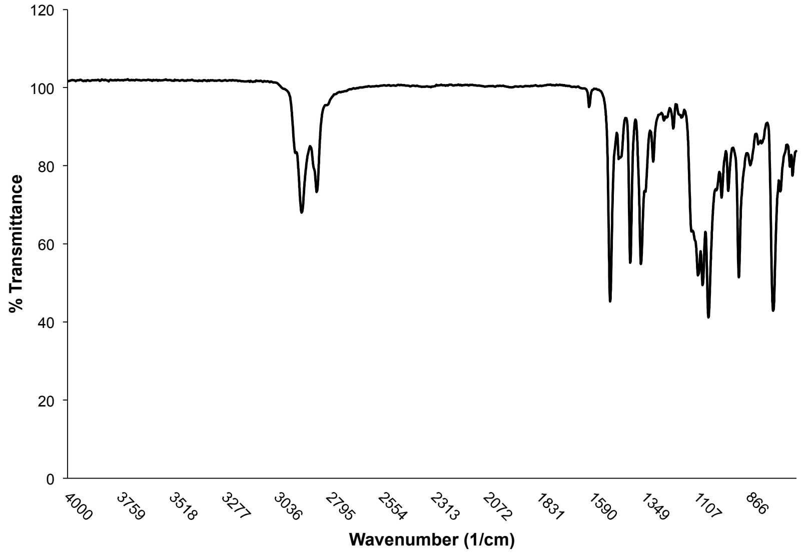

Figure **S35**: FTIR-ATR of Compound **7b** - 5,7-dibromo-2-((oct-7-en-1-yloxy)methyl)-2,3-dihydrothieno[3,4-*b*][1,4]dioxine

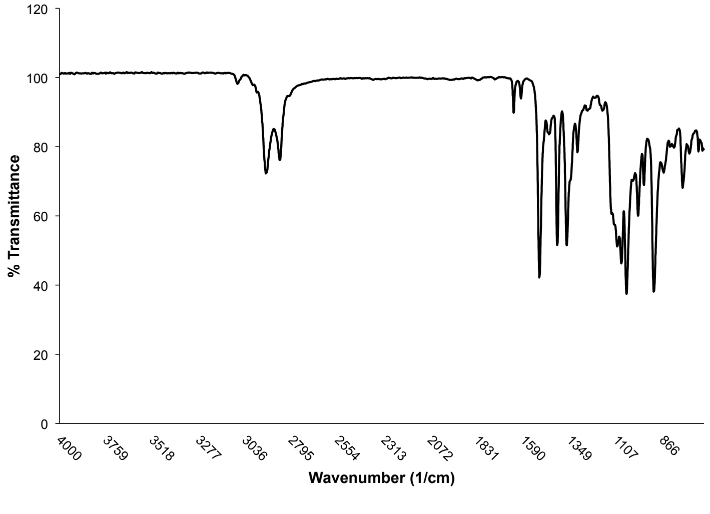

Figure **S36**: FTIR-ATR of Compound **P1**- poly(octenylEDOT)

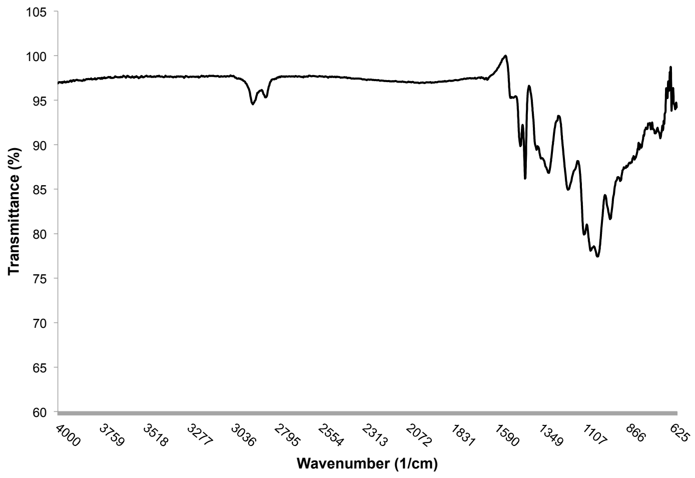

Figure **S37**: FTIR-ATR of Compound **P2**- Poly(octenyl-HFIP-PEDOT)

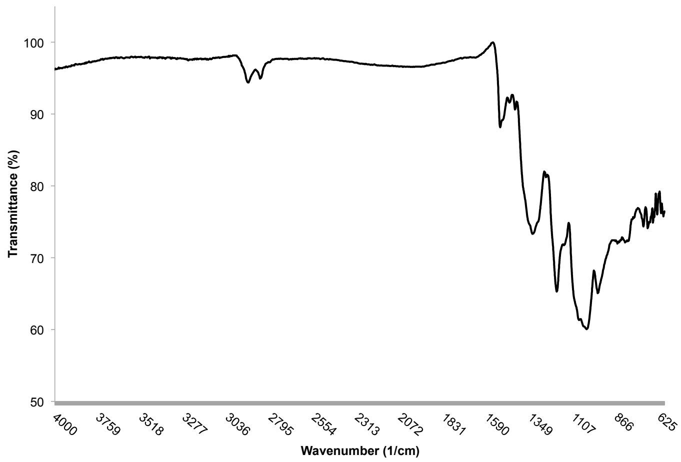

Figure **S38**: FTIR-ATR of Compound **P3**- poly(octyl-PEDOT)

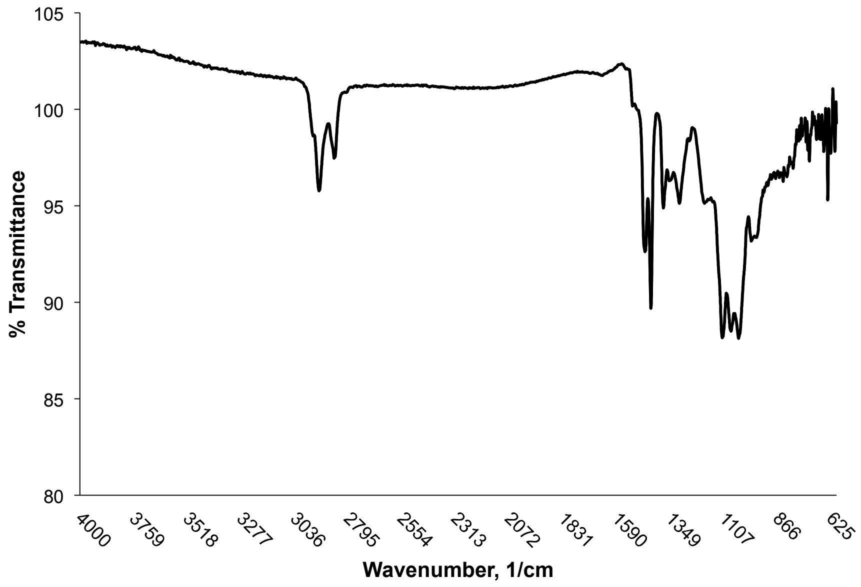

Figure **S39**: Schematic/cartoon of sensing experimental set up

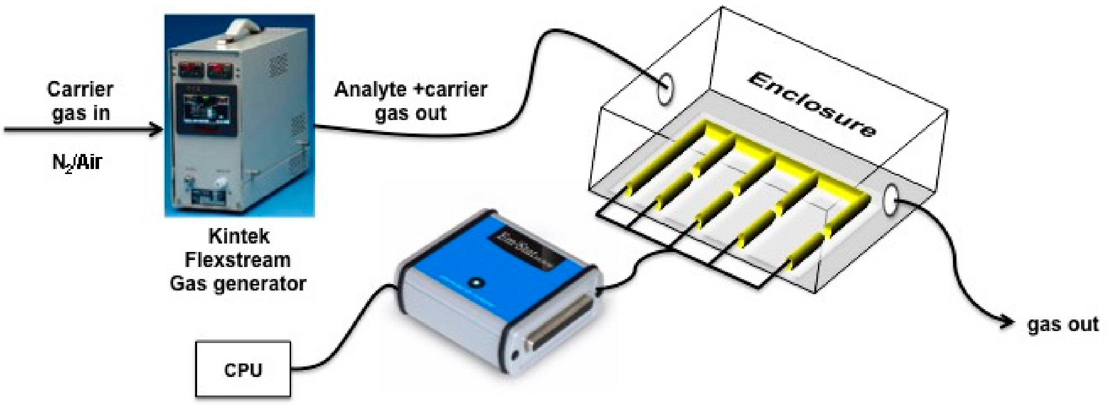

Figure **S40**: Photograph of sensing experimental set up

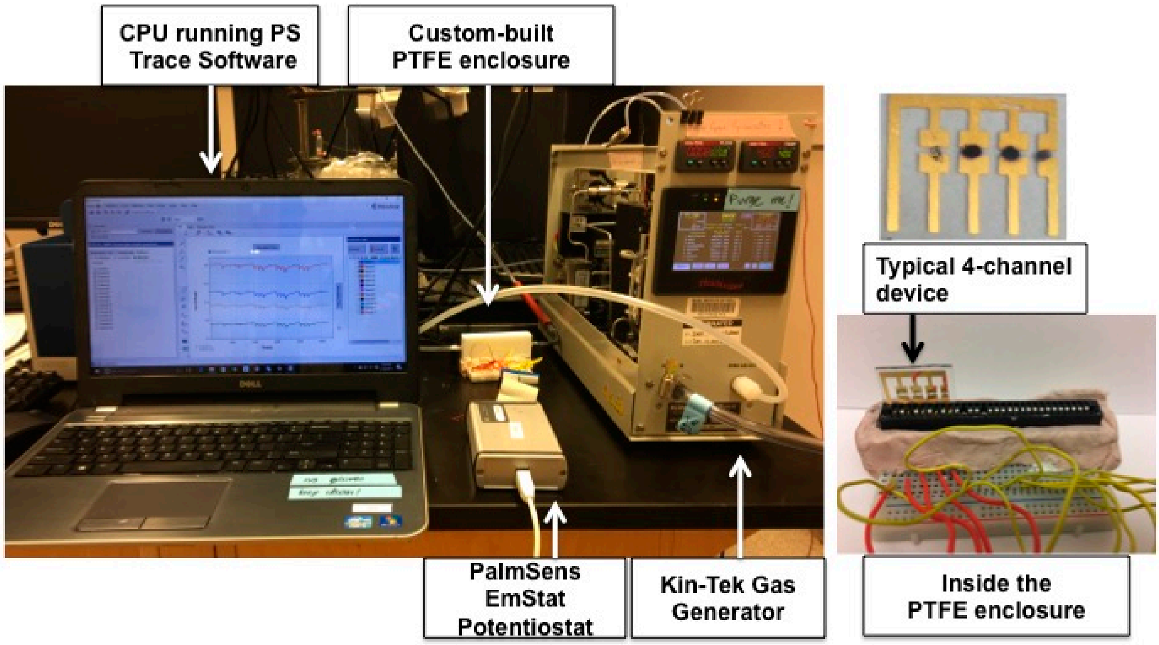

Figure **S41**: Schematic/cartoon of sensing experimental set up for sensing in humidified carrier gas (air)

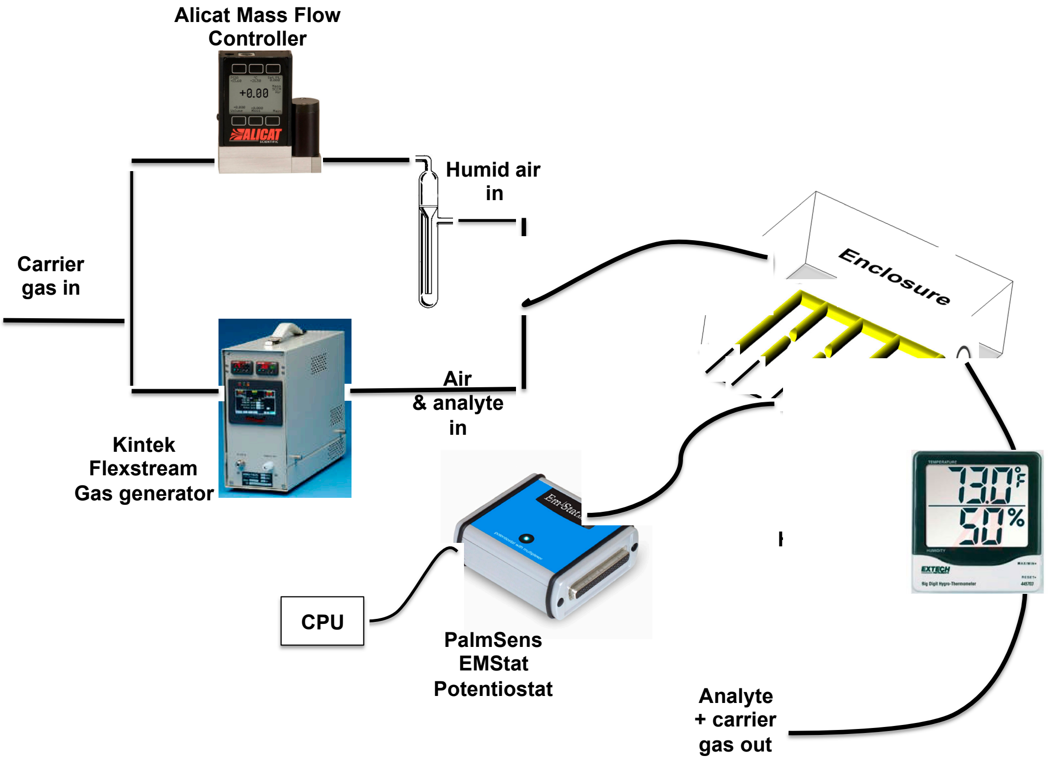

Figure **S42**. Chemiresistive response saturation times for **P2**/SWCNT in N_2 (blue triangles) and air (24% RH) (red diamonds) to increasing concentrations of DMMP(The legend for air data here is a square not a diamond)

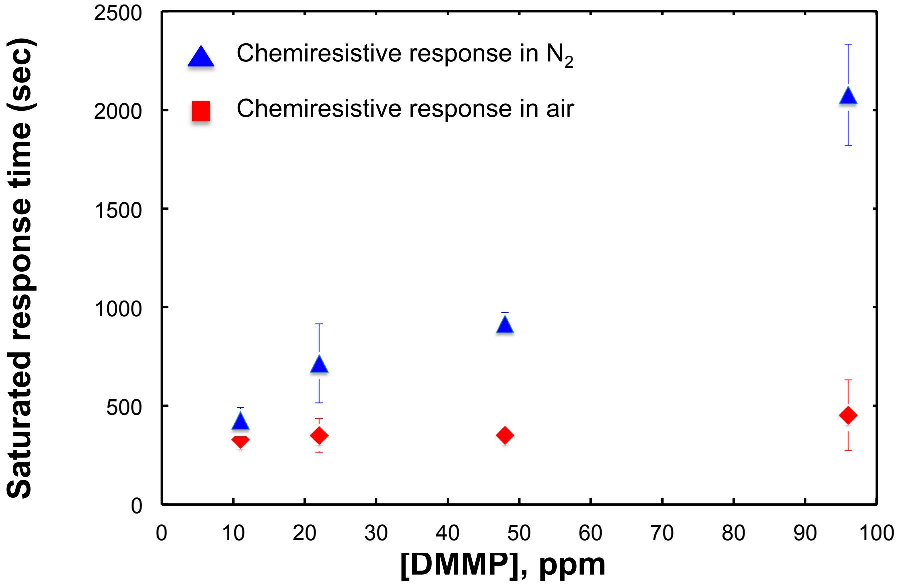

Figure **S43**. Linear response curves for **P2**/SWCNT in N_2 (blue triangles) and air (24% RH) (red diamonds) to increasing concentrations of DMMP

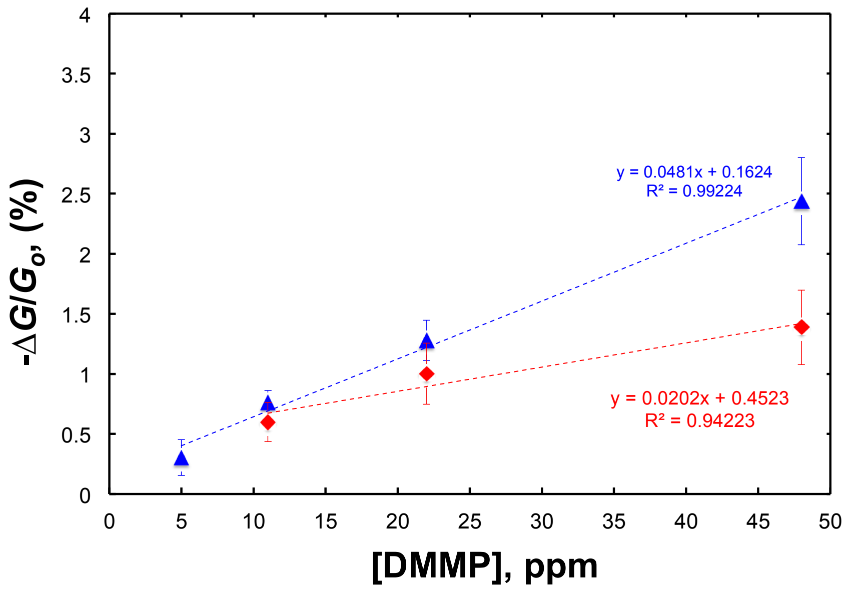

Figure **S44**. Chemiresistive response versus **P2**/SWCNT device age, in days. Devices were exposed to 11 ppm DMMP diluted with N_2 until saturation was reached, then stored under ambient conditions.

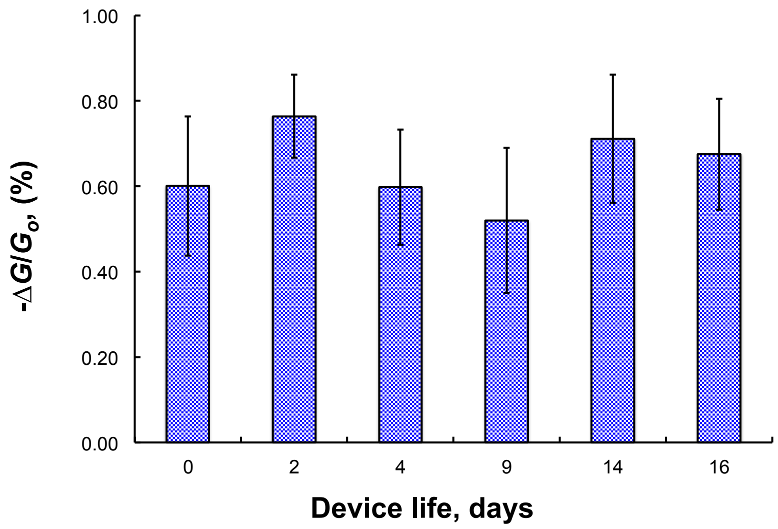

Figure **S45**. Conductance traces of three **P2**/SWCNT chemiresistors to 11 ppm DMMP in humid air (50% RH)

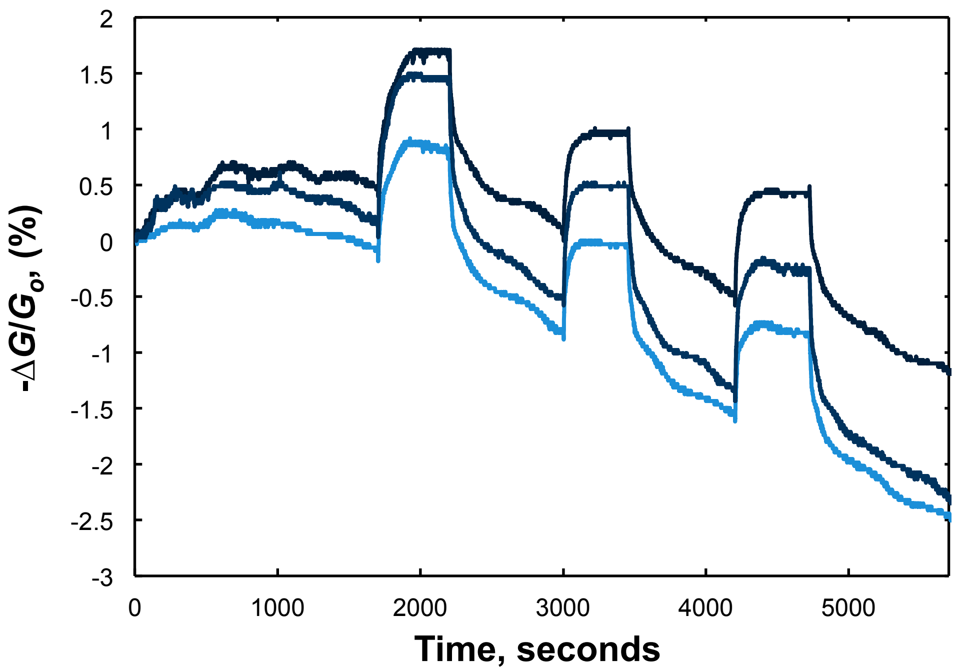

Supplement: Supplementary file 1 [file sensors-17-00982-s001.pdf]
